# Invisible Thermoplasmonic Indium Tin Oxide Nanoparticle Ink for Anti-counterfeiting Applications

**DOI:** 10.1021/acsami.2c10864

**Published:** 2022-07-22

**Authors:** Arianna Mazzotta, Alessio Gabbani, Marco Carlotti, Marina Ruggeri, Elvira Fantechi, Andrea Ottomaniello, Francesco Pineider, Andrea Pucci, Virgilio Mattoli

**Affiliations:** † Center for Materials Interfaces, Istituto Italiano di Tecnologia, Viale R. Piaggio 34, Pontedera 56025, Italy; ‡ The Biorobotics Institute, Scuola Superiore Sant’Anna, Viale R. Piaggio 34, Pontedera 56025, Italy; § Department of Chemistry and Industrial Chemistry, University of Pisa, Via Moruzzi 13, 56124 Pisa, Italy

**Keywords:** thermoplasmonics, indium tin oxide, heavily doped semiconductor nanoparticles, ink-jet printing, anti-counterfeiting

## Abstract

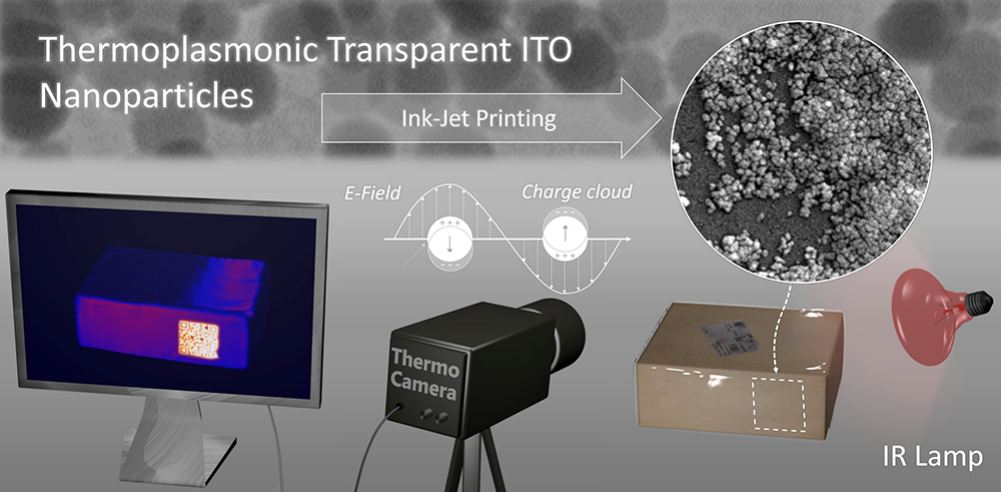

In this study, we present a thermoplasmonic transparent ink based on a colloidal dispersion of indium tin oxide (ITO) nanoparticles, which can offer several advantages as anti-counterfeiting technology. The custom ink could be directly printed on several substrates, and it is transparent under visible light but is able to generate heat by absorption of NIR radiation. Dynamic temperature mapping of the printed motifs was performed by using a thermal camera while irradiating the samples with an IR lamp. The printed samples presented fine features (in the order of 75 μm) and high thermal resolution (of about 250 μm). The findings are supported by thermal finite-element simulations, which also allow us to explore the effect of different substrate characteristics on the thermal readout. Finally, we built a demonstrator comprising a QR Code invisible to the naked eye, which became visible in thermal images under NIR radiation. The high transparency of the printed ink and the high speed of the thermal reading (figures appear/disappear in less than 1 s) offer an extremely promising strategy toward low-cost, scalable production of photothermally active invisible labels.

## 1 Introduction

Counterfeiting of goods, ranging from valuable documents, currency, and branded products up to commercialized production and human health, is a rapidly expanding issue in our society, leading to challenging problems with economic and safety impacts.^1^ Due to the recent development of high-resolution equipment, such as digital cameras, scanners, and printers, counterfeiters are able to produce high-quality replicas by simply using their personal computers.^2^ Such illegal activities not only harm the companies producing the goods, violating their rights, and leading to a loss of business but also pose high risks to the society. A clear example is the counterfeiting of drugs (by employing unknown and/or incorrect amounts of ingredients), which puts the health of patients at high risk.^3^ To prevent these events and fight counterfeiting, the development of technologies able to authenticate brands and documents is of fundamental importance.^2,4,5^ To date, the most common anti-counterfeiting methods use tags that can be easily cloned due to their low complexity and high predictability.^6^ However, technological advances made it possible to explore several solutions to the challenges posed by counterfeiting developing systems that are simple to fabricate but complex to replicate. Among these, one may find electronic systems like RFID tags^7,8^ or smart optical marking methods such as holograms,^9−11^ barcodes,^12−16^ box seals, watermarks,^17^ and luminescence printing.^5,18^ Recent works in the literature propose also physically unclonable functions (PUFs) to be used in the fabrication of security labels, exploiting some inherent random variations introduced by the manufacturing process. This aspect involves a very high level of security since the PUF system is impossible to copy and, consequently, only those who know the decryption key will be able to read the message.^19−21^

Notably, several anti-counterfeiting technologies – including some PUF-based systems^20,22,23^ – make use of nanoparticles (NPs) especially because of their unique and highly tunable physical properties, their responsiveness to several stimuli, and the possibility to integrate them into devices through solution processing.^24^ Compared to molecular technologies, nanoparticle-based approaches are more complex to counterfeit since their properties are highly dependent on the synthetic method, composition, and interfacial chemistry and thus cannot be easily reverse-engineered.

Of particular interest is the use of NPs as substrates capable of interacting with light (through phenomena such as fluorescence^25,26^ or upconversion)^27−29^ to display on-demand a specific fabricated pattern as a visible readout signal. Moreover, plasmonic NPs exhibit increased light absorption when irradiated at their specific resonant frequency, which is tunable with size, shape, and material composition. The absorbed energy can then be released thermally, with the NPs acting as a nanosource of heat that can be remotely and rapidly activated by light.^30,31^ In recent years, this emerging field called thermoplasmonics, initially applied to biomedicine^32,33^ and photocatalysis,^34^ has started to be employed in anti-counterfeiting technology. To this aim, plasmonic NPs able to generate heat upon absorption of infrared radiation while maintaining transparency under visible light are desirable. A recent example of the application of thermoplasmonics in anti-counterfeiting technology can be found in the work by Kang *et al.*, where the authors used inks comprising gold nanorods and nanospheres to absorb near infrared (NIR) and visible light, respectively, and generated different heat patterns depending on the wavelength of the illuminated light. The heat generated upon irradiation at the NIR plasmonic resonance was used as the optical readout with a thermal camera while irradiation with visible light exposes a different pattern.^35^ However, Au nanorod inks are not fully transparent under visible radiation as they also absorb red light due to the excitation of the transverse plasmon resonance along the short axis of the rod. In this respect, fully transparent inks would be more difficult to identify and counterfeit.

Beyond metallic nanostructures, heavily doped semiconductors, with relatively high free carrier densities, are emerging as plasmonic nanomaterials with a tunable resonant frequency spanning from the visible up to the far infrared range.^36−41^ Among these, indium tin oxide (ITO) is intrinsically transparent in the visible range due to its wide band gap while the controlled aliovalent substitution of In^3+^ ions with Sn^4+^ dopants generates the free electrons needed to support a plasmonic resonance in NPs, thus inducing the strong absorption of infrared light. Furthermore, colloidal ITO NP dispersions can be produced in relatively large amounts by chemical methods, such as decomposition of organometallic compounds^42^ or solvothermal methods.^43^ Compared to traditional noble metal plasmonic NPs, ITO NPs offer several advantages. First, they are cheaper and suffer only moderately from the oxidation damage to which many metals are prone. In addition, shifting the plasmonic resonance in the infrared for noble-metal NPs requires efforts in shape engineering to obtain rods or complex anisotropic nanoarchitectures,^44−47^ which are thermally unstable and thus can be subjected to reshaping upon thermal heating or long-term aging.^48^ On the other hand, in ITO NPs, infrared plasmonics is achievable in simple spheres, which are easier to synthesize and whose morphology is thermally stable. Moreover, given the long wavelength at which they exhibit plasmonic properties, Mie scattering is further reduced in these nanostructures, and absorption is dominating, which is the crucial contribution to convert photons into heat.^49^ All these features make colloidal dispersions of ITO NPs very attractive as anti-counterfeiting inks exploiting the thermoplasmonic effect upon infrared light illumination.

In this work, we propose a novel anti-counterfeiting approach based on the direct printing of a plasmonic ITO NP-based custom ink to obtain arbitrary patterns with high resolution (250 μm), which are invisible to the naked eye but evident when exposed to NIR radiation.

The printing process is enabled by inkjet-printing technology as it offers multiple notable advantages such as low-cost production and unconstrained pattern design ability and it is compatible with various ink formulations^50^ as well as supporting substrates of diverse materials such as glass, polycarbonate, paper, and polyethylene naphthalate (PEN) reported in this work.^22,28,50,51^ We show that the printed ITO films are highly transparent and that they selectively respond to NIR radiation fast (<1 s) and reliably. Finally, we show the application of the methodology herein presented for the preparation of an anti-counterfeiting prototype based on an only-NIR visible QR code, which can be also placed on top of a traditional one and selectively read through an IR thermal camera. Remarkably, the readout could be performed using standard IR cameras and cheap illumination sources (such as broadband infrared lamp) instead of NIR lasers, making this anti-counterfeiting technology easy to implement for many different applications and environments.

## 2 Results and Discussion

### 2.1 Synthesis and Characterization of the NP Dispersion

**Figure 1:**
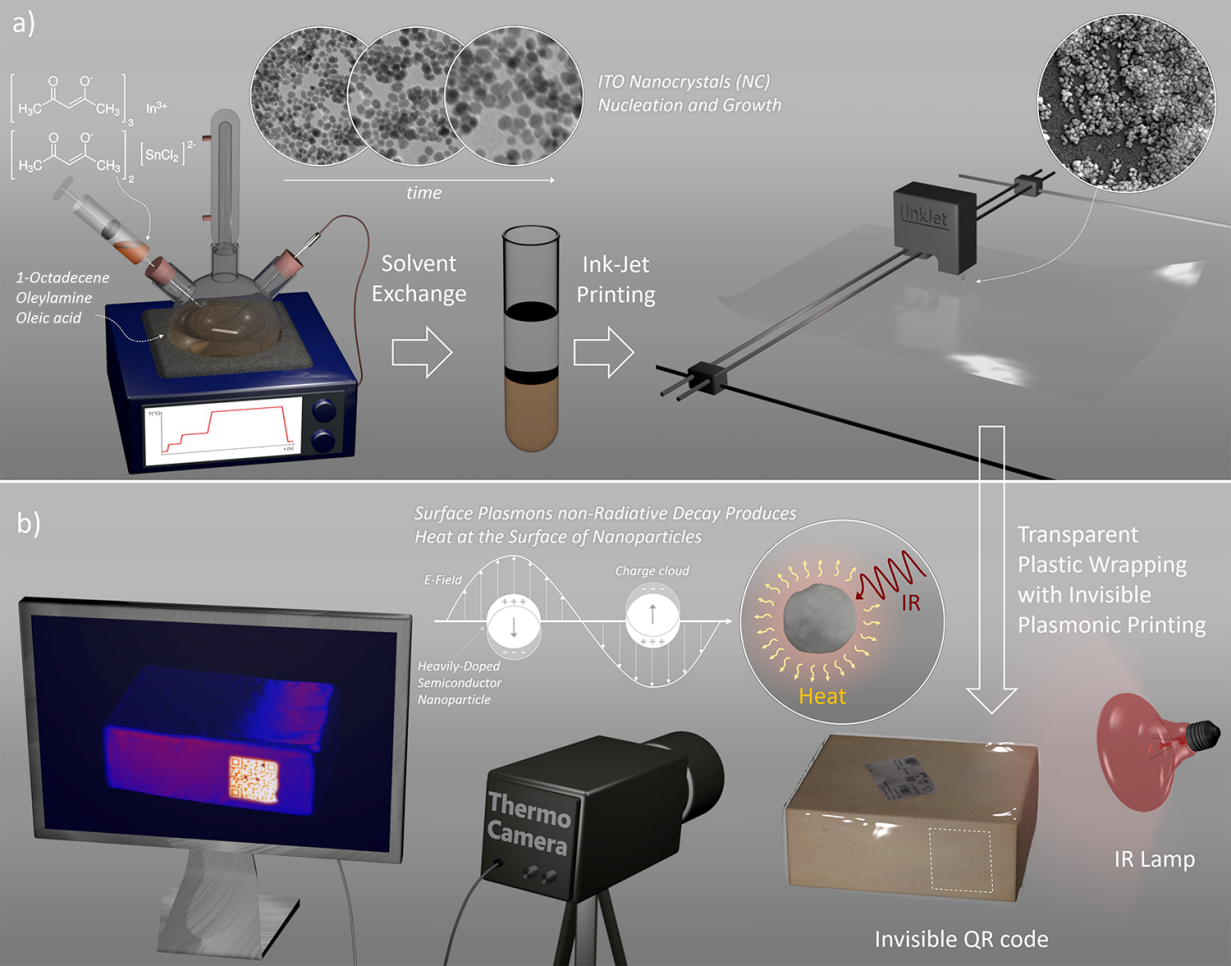
(a) Scheme of ITO NP ink synthesis and application. (b) Concept of thermoplasmonic labeling for anti-counterfeiting applications.

The aim of the present study was to develop a novel anti-counterfeiting technology based on barcodes visible only if irradiated under NIR light. To do so, we developed a printable ink comprising a colloidal dispersion of ITO NPs featuring a plasmon resonance in the NIR part of the light spectrum while remaining transparent in the visible range (Figure 1b).

The dispersion of ITO NPs was prepared using a bottom-up strategy involving the thermal decomposition of organometallic precursors (indium acetylacetonate, In(acac)_3_, and tin bis(acetylacetonate) dichloride, Sn(acac)_2_Cl_2_) in different ratios and in the presence of surfactants (oleic acid and oleylamine) and in a high boiling point organic solvent as depicted in Figure 1a. More details on the synthesis are provided in the Methods section. The developed synthetic strategy, inspired by literature approaches,^42^ provides excellent control over the morphology, doping content, and average size of the NPs as well as good crystallinity of the material, involving a simple heat-up approach and avoiding more complicated and time-consuming multistep procedures such as the controlled-injection approach.^52^

Tin doping of the NPs and an inert atmosphere in the synthesis are necessary to obtain the localized surface plasmon resonance in ITO nanocrystals.^53,54^ Several batches of ITO NPs have been prepared, containing different amounts of tin dopant ranging from 0% (i.e., nanocrystalline In_2_O_3_ without dopant, denoted as ITO-0 for consistency) to 15% (i.e., ITO-15). The concentrated NP dispersions (about 30 mg/mL) displayed a light blue-green color, slightly varying with the composition. This is ascribed to the tailing of the plasmonic resonance as shown in the extinction spectra (see Figure S1), which at such high concentrations is extended up to the visible part of the spectrum due to the intense absorption coefficient in the NIR. The diluted samples (about 70 μg/mL concentration) used to collect the NIR absorption spectra are transparent to the naked eye.

Complete characterization of all the produced batches is reported in the Supporting Information (Figures S1–S3, Tables S1 and S2). Noteworthily, the possibility of easy tuning of the absorption peak by finely controlling the dopant concentration can be of great practical interest toward specific and tailored technological applications in the NIR range. At high doping levels, the ability of Sn^4+^ dopant to provide free electrons decreases. This is ascribed to the formation of irreducible complexes between the tin cations and interstitial oxygen atoms, hindering the generation of free electrons.^54,55^ As a consequence, above a threshold of 10% doping, additional incorporation of Sn^4+^ does not shift the plasmonic resonance to higher energies (Figure S1). Moreover, in this regime, the further addition of Sn cations is known to broaden the resonance and decrease the plasmonic quality factor due to impurity scattering.^54^ This is also confirmed in our series by the broadened extinction peaks of the highly doped samples (ITO-12 and ITO-15, see Figure S1). To identify the most efficient sample in absorbing NIR photons, we estimated experimentally (more details can be found in the Experimental Methods section) the extinction coefficients of ITO-2.5, ITO-5, and ITO-10, which were 13.5, 18.7, and 20.0 μm^–1^ respectively, in good agreement with the data reported by Staller *et al.* for NPs of comparable size and doping amount.^56^ It can thus be argued that the 10% doped sample (ITO-10) displays the highest extinction coefficient and the sharpest plasmonic resonance among the samples of the series. For this reason, we focused our attention to ITO-10 for the development of the anti-counterfeiting ink.

**Figure 2:**
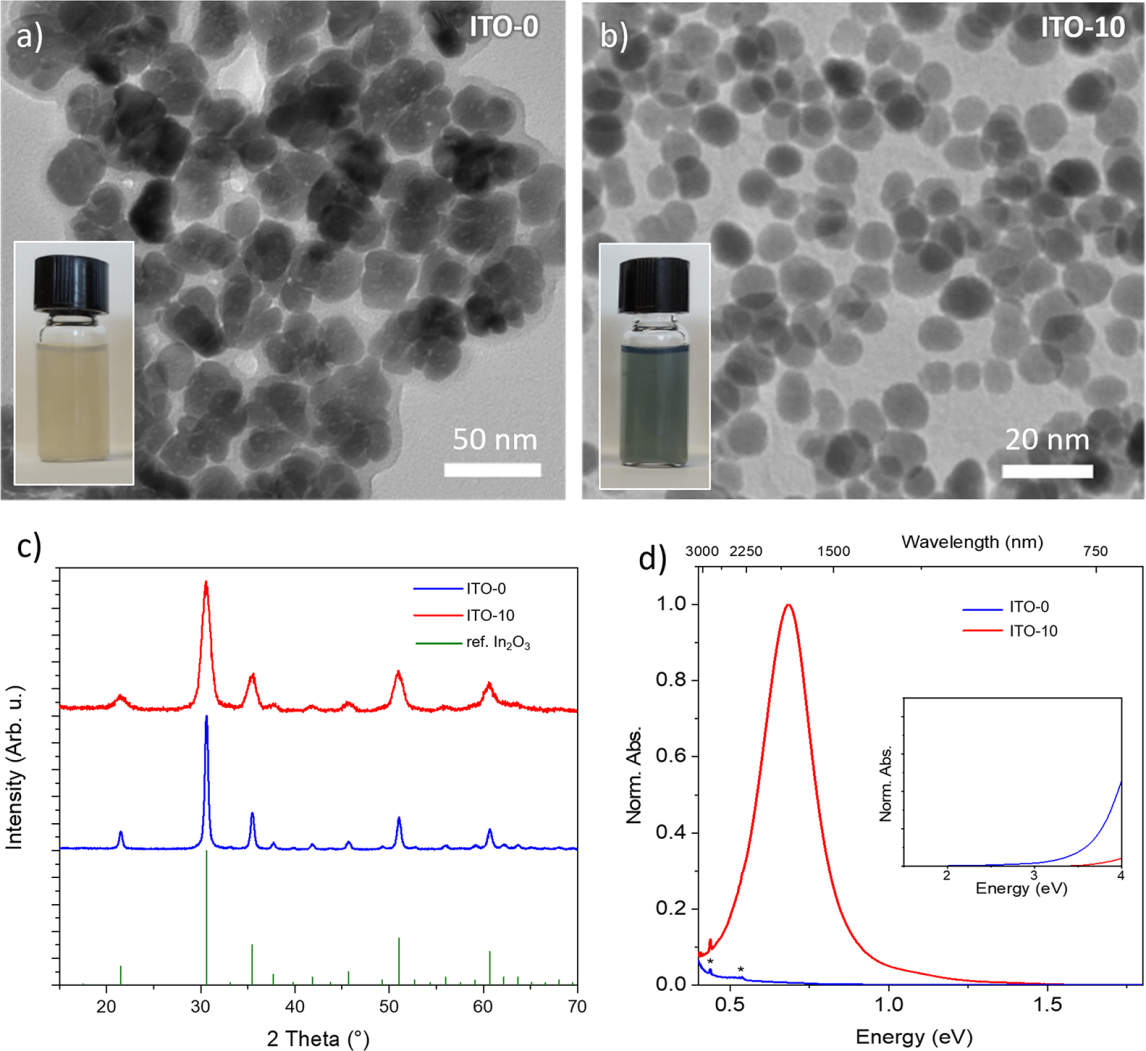
TEM images of ITO-0 (pure In_2_O_3_) (a) and 10% doped ITO NPs (ITO-10) (b). The insets show images of highly concentrated samples. (c) X-ray diffraction pattern of ITO-0 and ITO-10 NPs samples, compared to the reference pattern of In_2_O_3_. (d) Absorption spectrum of ITO-0 and ITO-10 NPs dispersed in C_2_Cl_4_. The asterisks mark vibrational signals due to the oleic acid coating. The inset in panel (d) highlights the visible region of the spectrum.

TEM investigation revealed quasi-spherical NPs for all the different compositions (see Figure S2). The average size of undoped ITO-0 NPs is 21 ± 4 nm (Figure 2a), while the doped ones are smaller. For instance, ITO-10 NPs show an average diameter of 9 ± 2 nm (Figure 2b) (detailed characteristics of other formulations can be found in Table S1). A single cubic crystalline phase was observed through powder X-ray diffraction (XRD) in both samples (Figure 2c), with a lattice parameter consistent with the reference pattern of In_2_O_3_ (10.118 Å) and negligible changes in the Sn-doped NPs. The coating of a surfactant layer (∼8 wt %, evaluated through thermogravimetric analysis) confers a high colloidal stability on nonpolar solvents. The Sn incorporation in the ITO NPs perfectly matches the initial precursor ratio (Table S1), as detected through inductively coupled plasma atomic emission spectroscopy (ICP-AES). Such effective incorporation of aliovalent dopant cations is crucial to introduce free electrons in semiconductor NPs,^57,58^ and it is responsible for the sharp plasmonic resonance at 0.68 eV (1800 nm) in ITO-10, which is absent in undoped In_2_O_3_ (Figure 2d). Remarkably, a high transparency to visible radiation is displayed for both samples due to their wide band gap (3.6 and 3.8 eV, respectively, for ITO-0 and ITO-10).

### 2.2 Thermo Ink Preparation and Printing

**Figure 3:**
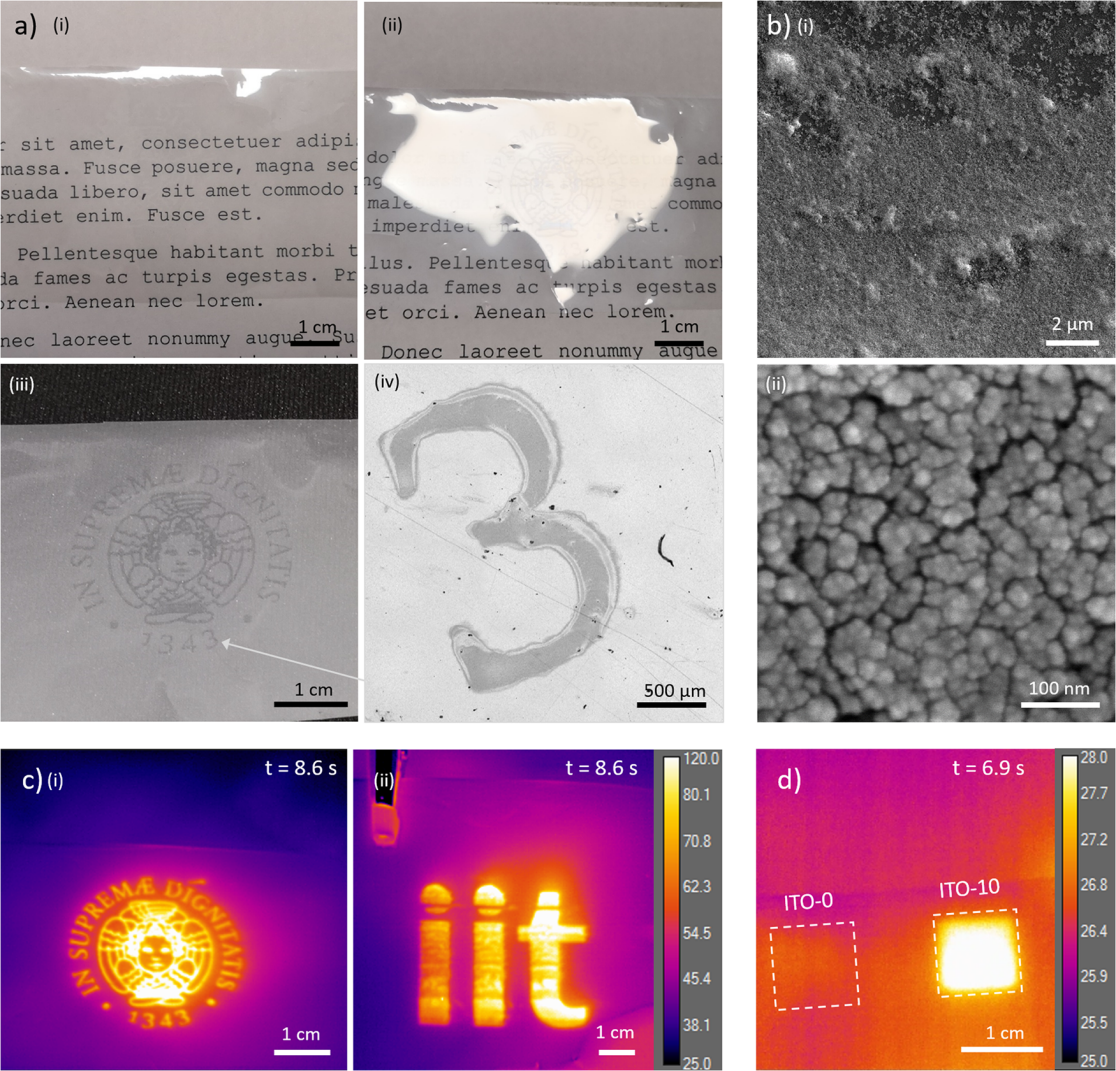
(a) Optical image of a printed Cherub, the logo of the University of Pisa, at different illumination conditions: (i) random orientation (most of the cases, invisible pattern), (ii) light source and camera in a specular position at 30° with respect to the plane of the sample, (iii) diffused light source and camera in a specular position at 30° with respect to the plane of the sample with a dark background; (iv) optical microscopy of a detail of the printed sample highlighting the quality of the ITO ink print. (b) Scanning electron microscopy (SEM) images at various magnification levels of the printed ITO NPs on PEN substrate. (c) Thermal image of the Cherub sample (i) irradiated with an IR lamp (100 W@20 cm); thermal image of the IIT logo (ii) also printed on the PEN substrate, irradiated in the same condition. (d) Thermal imaging of printed ITO-10 (doped) and ITO-0 (undoped) NPs, under IR irradiation (100 W@50 cm): ITO-0 sample does not show a significant temperature increment with respect to the substrate. Timestamp in panels (c) and (d) indicates the time elapsed from the beginning of IR irradiation.

The ITO-10 dispersion described above was used as a plasmonic ink that was loaded into the inkjet printer allowing the fabrication of multiple patterns that were transparent to the naked eye but thermally visible under NIR irradiation. In particular, after the solvent exchange (from hexane to dichlorobenzene) to optimize the printing process, we used the prepared dispersion at a nominal concentration of 36 mg/mL to directly deposit ITO NP layers by inkjet printing it on a target substrate of TEONEX PEN film. The latter was selected due to its transparency to visible and NIR radiation, flexibility, and low thickness (∼25 μm, which enhances the resolution during readout, as we will discuss later). Figure 3a(i–iii) highlights how the printed patterns were visible only in particular light conditions (light source and viewpoint in specular position with respect to the plane of the sample, with homogeneous dark background) due to the inevitable Rayleigh scattering of UV–Visible wavelengths by the NP aggregates formed during the deposition.

The sample representing the Cherub, the logo of the University of Pisa, appeared clearly visible only when placed on a black surface and observed through a camera oriented at a particular angle to maximize the reflected light over the scattering (see Figure 3a(iii)), while under normal observation conditions, it is mostly transparent or not clearly visible (Figure 3a(i,ii)). Moreover, bright field microscopic images of the same Cherub sample show that it is possible to print high-resolution letters and numbers as well as precise designs as shown in Figure 3a(iv) (see also Figure S4). The high-magnification SEM images reported in Figure 3b show how the ITO NPs adhere to the PEN substrate to form a continuous and uniform layer.

Under irradiation with an IR lamp, the features of the printed images became clearly visible on a thermal camera as we show in Figure 3c, where we reported the steady-state response of the aforementioned Cherub (dimensions: 3 × 3 cm) and of the Italian Institute of Technology logo (dimensions: 5 × 4.5 cm) (both reaching up to 120 °C under the operating conditions). These figures show how the novel ITO NP ink can be used in inkjet printers to realize complex and fine designs with consistent thermal properties. Notably, no heating effect was obtained when we employed ITO-0 NPs (Figure 3d), supporting the hypothesis that the generation of heat is triggered by the excitation of the surface plasmon resonance and highlighting the importance of Sn doping to generate such an optical feature.

**Figure 4:**
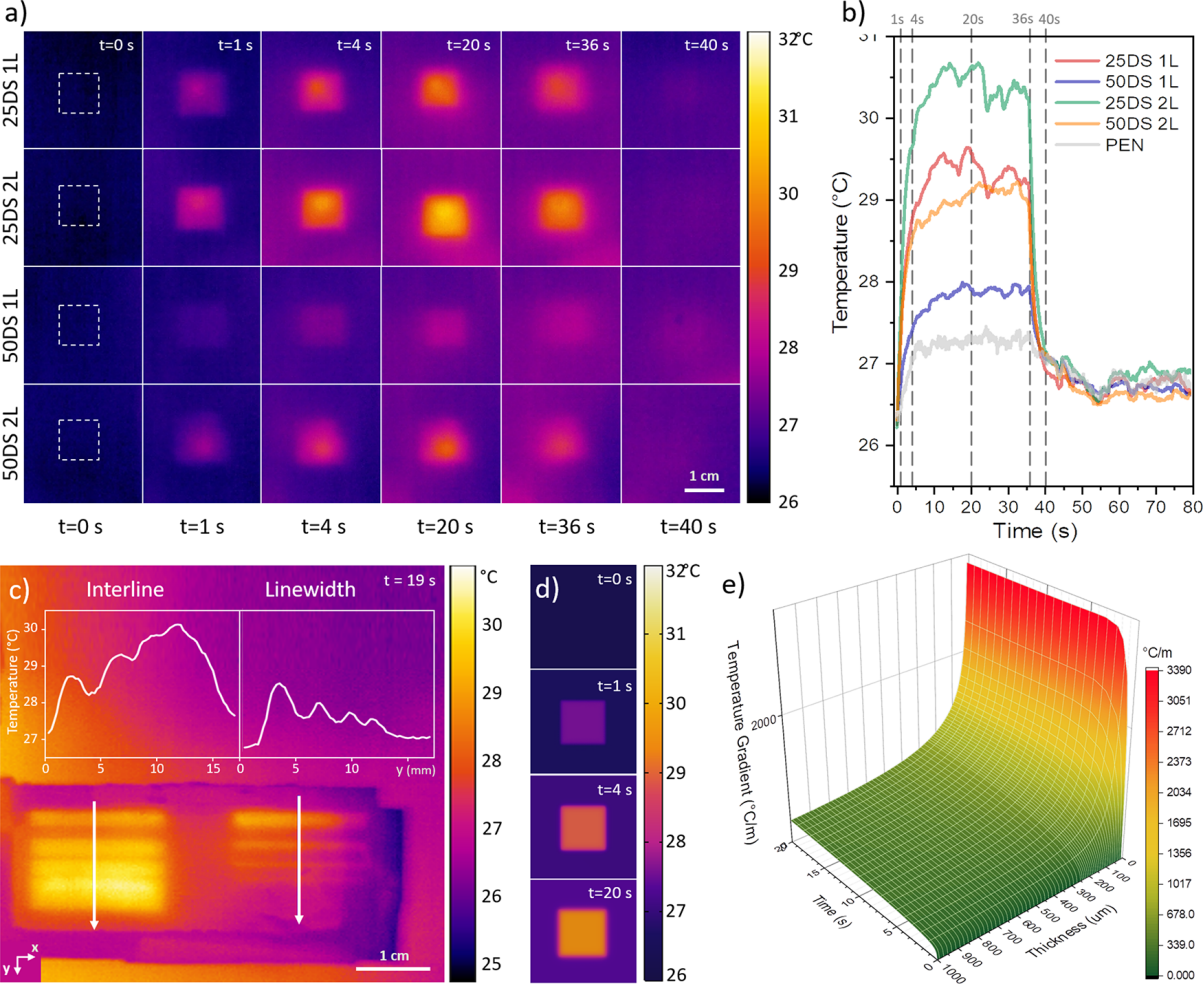
(a) Thermal images of ITO-10 NP samples printed with different parameters, exposed to IR lamp irradiation (100 W@50 cm), acquired ad different times, as in panel (b); sample 25DS 1 L, 25DS 2 L, 25DS 2 L, and 25DS 2 L refer to various combinations of drop spacing (DS = 25, 50 μm) and number of printed layers (L = 1, 2). (b) Thermal dynamics of the same samples, averaged with an ROI (region of interest) of 0.5 × 0.5cm^2^ placed in the center of the printed squares. Irradiation with the IR lamp starts at 0 s and ends at 36 s. (c) Evaluation of thermal pattern resolution considering the two different designs “interline” and “linewidth”, performed on a specific printed sample exposed to the IR lamp (100 W@50 cm): interline on the left has widths of 2500, 1000, 500, and 250 μm (from top to bottom); linewidth on the right has widths of 2500, 1000, 500, and 250 μm (from top to bottom). (d) Simulated thermal image of the 25DS 1 L square sample (1 cm side) obtained by finite element modeling (FEM) Multiphysics software. (e) Thermal resolution, evaluated as the gradient of temperature along the *x* axis at the edge of the ITO square vs substrate thickness and time, obtained by simulation. Timestamp in panels (a), (c), and (d) indicates the time elapsed from IR irradiation starting.

To better characterize the printing performances, we investigated how different parameters – e.g., drop spacing (DS; i.e,. center-to-center distance from one drop to the next in the *X* and *Y* positions) and the number of printed layers – can influence the final properties of the print in terms of resolution, transparency, absorbance, and effectiveness of the plasmonic photothermal effect. To this aim, we prepared samples employing two different DS values of 25 and 50 μm (corresponding to a resolution of 1016 and 508 dpi) and two different numbers of printed layers (one and two). We used printed square samples (1 × 1 cm^2^) to characterize the photothermal activity and printed lines with a 250 μm nominal width to measure the thickness of the printed ITO-10 ink at various printing parameters. Stylus profilometry measurements showed consistent results revealing an increased thickness for samples printed using a lower DS and/or a higher number of layers, two conditions which increase the number of particles printed per unit area (Figure S5). Optical transmittance measurements in the visible range made on square samples showed that the deposition of ITO NPs to print the figures did not affect significantly the transparency of the substrate (Figure S6), with an about 1% decrease of transmittance for all the parameters tested. To investigate the photothermal activity of the ITO NP layers, we exposed the printed squares to an infrared lamp (100 W IR lamp, placed at a 50 cm distance from the sample, providing an irradiance of 28 mW/cm^2^ in the perpendicular projection point) and recorded the temperature using an IR thermal camera. The measured heating dynamics are shown in Figure 4a,b.

As the lamp was turned on, the temperature sharply increased and the squares became visible in the thermal images in less than 2 s. Conversely, when the lamp was turned off, the samples rapidly cooled down until reaching the ambient temperature and the printings disappeared, leaving the effect of a blank PEN. Remarkably, the thermal response was proportional to the nominal density of NPs in the print. The square obtained with two layers printed at 25 μm DS reached a higher temperature compared to the others and with faster kinetics (initial heating of 1.9 °C s^–1^ with Δ*T* = 4 °C) while the one comprising one layer and 50 μm DS showed a weaker response (0.5 °C s^–1^ and Δ*T* = 1.4 °C). The other two samples (one layer, 25 μm DS and two layers, 50 μm DS) behaved similarly in terms of heating efficiency (1.5 °C s^–1^), showing almost the same maximum temperature reached in the steady state (Δ*T* = 2.9 ± 0.2 °C) (see also Figure S7). On the other hand, the cooling dynamics were comparable for all the samples. We can ascribe this observation to the small thickness of the printed layer (about a hundred times smaller than the substrate), which does not contribute to thermal inertia. In particular, the exponential decay constant was in the range 1.5 ± 0.3 s for all the samples (see Figure S7d).

Based on these results, a DS of 25 μm and deposition of a single layer (DS25 1 L) were chosen as optimal printing parameters for the fabrication process, as they resulted in a good tradeoff between the amount of deposited material (recognizable shapes, good resolution), fabrication time, and sufficient thermal response to make the figures distinguishable through a thermal camera.

To quantitatively evaluate the plasmonic absorption characteristics of the printed ITO NPs, the IR absorbance spectrum of a printed ITO-10 sample was acquired and further analyzed. Noteworthily, the absorbance of the nanometric thin ITO film at the absorption peak is about 0.52, corresponding to the absorption of about 70% of the incident light. Compared to that in diluted dispersion, the absorbance spectrum of the printed film shows a significant redshift of the main peak (from about 1600 to 2200 nm) and an increase of its linewidth (from about 500 to 1000 nm) as shown in Figure S8. We ascribed this to the increase of the local refractive index of the medium that surrounds each NP, as predicted by the Maxwell–Garnet effective medium approach at a high volume fraction of NPs, and possibly the dipolar interactions between NPs, occurring when the interparticle distance is very small (see discussion in the Supporting Information). Both spectra (ITO NP dispersion and film) can be well fitted with consolidated models (i.e., Maxwell–Garnet effective medium approach, further details in Supporting Information – Modeling and Fitting of ITO Nanoparticle Dispersions and Solid Film), obtaining highly consistent results (see Figure S10).

Since the main application of the ink proposed herein is anti-counterfeiting, a study of the spatial resolution is beneficial to assess the possibility of printing barcodes for security labels of arbitrary dimensions. To assess this point, we first investigated the maximum resolution achievable while printing a single line. With our ink formulation and using a professional inkjet printing system (Dimatix DMP- 2800 printer), it is possible to obtain lines with widths down to 70 μm (see Figure S11). We then examined the thermal resolution of the lines by studying the thermal response of two different patterns, one consisting of straight lines of different widths with a fixed separation (called “linewidth” in Figure 4c) and one comprising straight lines of equal width but a different separation (called “interline” in Figure 4c). These two designs were aimed at understanding the minimum printable width and the maximum lateral resolution as well as their thermal detectability. In these experiments, the temperature profile averaged along five vectors crossing all the lines was considered, as shown in Figure 4c. The minimum linewidth was determined as the smallest width remaining distinguishable.

The achieved resolution allowed the thermal detection of printed patterns with a width below 250 μm. Similarly, the interline thermal resolution was evaluated as the smallest distance at which two lines remain separated from each other in the second design, which we evaluated to be between 250 and 500 μm. Even if these resolutions are more than satisfactory to realize barcodes of common sizes, we further investigated this aspect by means of finite-element method (FEM) simulations, in order to evaluate the influence of substrate thickness and exposure time on the achievable thermal contrast and thus on the thermal printing resolution.

### 2.3 Thermal Simulations

We implemented a 3D heat transfer model in COMSOL Multiphysics v5.6 in order to investigate the achievable thermal resolution as a function of substrate thickness and exposure time. The simulation model included the PEN substrate with a square-shaped area made of ITO NP dispersions at its center with the same geometrical parameters used in experimental tests (as in Figure 4a), considered as reference. We performed a time-dependent study whose details are available in the Experimental Section and in the Supporting Information. The model was validated by comparing the acquired data (dynamic profile of printed sample 25DS 1 L and PEN substrate, as in Figure 4b) with the simulation conducted in equivalent conditions, obtaining good matching as shown in Figure 4d (and more extensively in Figure S12). As input for the model, in addition to knowing the physical and material parameters, we used the experimentally acquired absorbance spectrum of the printed sample to estimate the fraction of the light power flux absorbed by the ITO film.

Once validated the model, we studied how the thermal resolution of the printed sample could be affected by the thickness of the substrate and the related time evolution, as they are the two main free parameters to be considered for real applications. As a meaningful indicator to estimate the thermal resolution achievable, we considered the gradient of temperature along *x* axis  on the edge of the ITO square (where the gradient maximum value is located). The temperature gradient is a good indicator of the theoretical achievable thermal resolution. In fact, by fixing a minimum detectable difference of temperature Δ*T*_min_, the theoretical minimum detectable features Δ*x*_min_ (resolution) can be estimated as Δ*x*_min_ ≈ Δ*T*_min_/. Thus, the higher the gradient, the higher (smaller in dimension) the resolution will be.

From the simulation, it is clear that the temperature gradient, once the steady state-condition is reached, is higher for thinner substrates, with respect to thicker ones. Moreover, the temporal dynamics are favorable for thinner substrates, reaching the thermal equilibrium much faster (see Figure 4e). This is particularly relevant for applications since thinner substrates will show a higher thermal contrast and shorter reading time.

### 2.4 Anti-Counterfeiting Demonstrative Application

**Figure 5:**
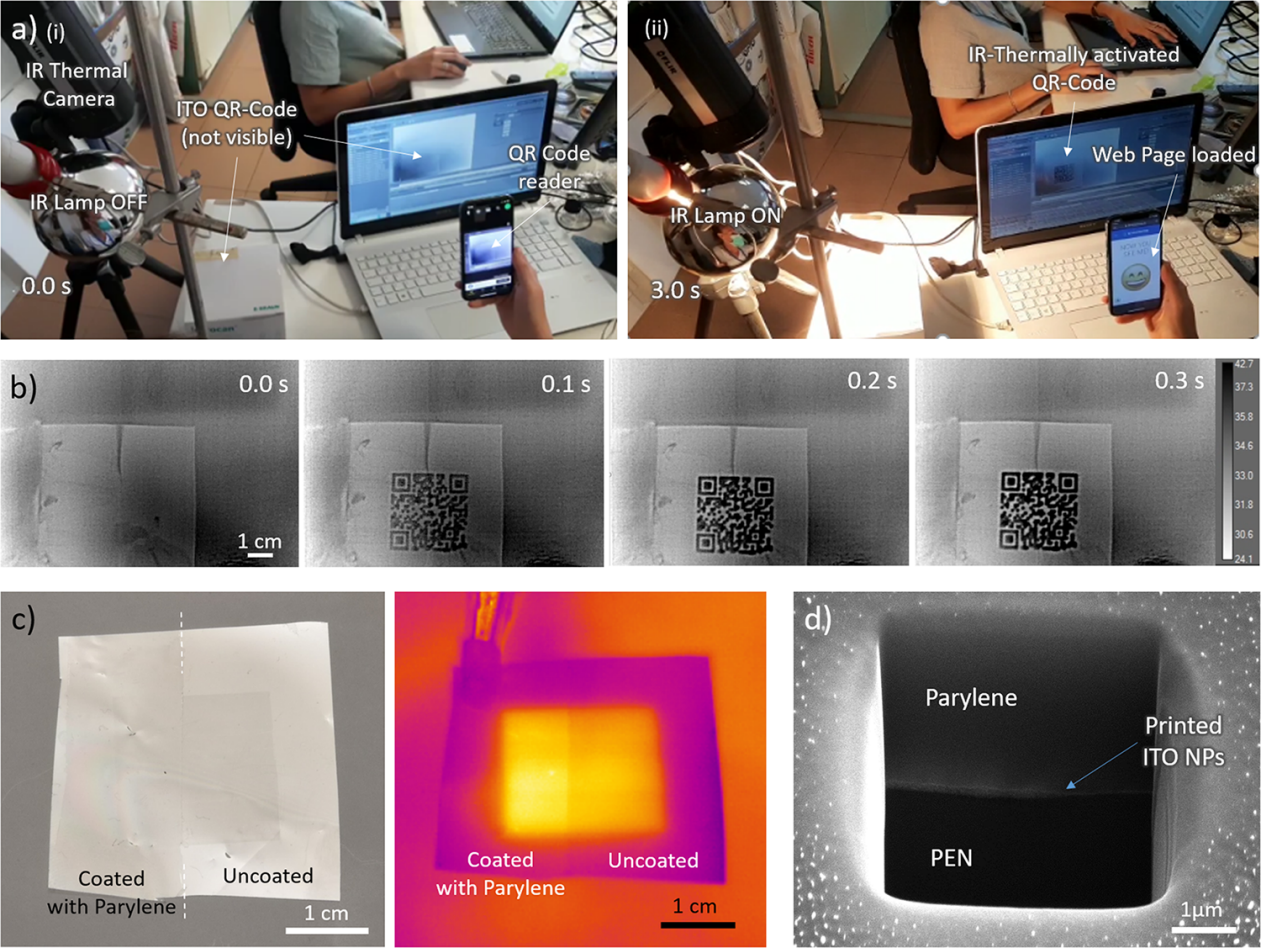
(a) ITO NPs printed invisible QR code demonstration (i). The QR code is linked to a specific webpage; (ii) when the code was irradiated with a NIR lamp (300 W@50 cm) and observed through an IR thermal camera, it became visible on the screen and the smartphone could recognize it, loading the target web page. (b) Details of the QR code thermal imaging dynamics: the QR code became visible in less than 300 ms. (c) (left image) Reduction of the reflex contrast of printed sample by using parylene coating post-process (left half of the sample covered) and (right) the effect of irradiation on thermal response. (d) SEM image (tilt angle 52°) of the focused-ion beam (FIB) section of the ITO NP printed sample on PEN and coated with 3 μm of parylene.

To evaluate the actual performance of this technology in a realistic scenario, we decided to test it on a QR Code, one of the most popular types of barcodes commonly used.^59^ Hence, we generated a QR code linked to a specific webpage, and we inkjet-printed it as a 3 × 3 cm^2^ image. When the code was irradiated with a 300 W NIR lamp and observed through an IR thermal camera as described earlier, it was mostly instantaneously (i.e., about 300 ms) visible on the computer screen and, in this way, it was possible to scan it simply using a smartphone (see Figure 5a,b and Video S1). The reading of the printed QR code was fast and reliable: the actual printing procedure took less than 10 min; considering the generation of the code, the preparation of the substrate, and the setting of all the thermal instruments for the reading, the whole procedure took less than 2 h. This suggests that the process could be easily scaled-up to meet production requirements. Moreover, it is possible to read the “invisible” QR code even when placed over a traditional QR code that did not absorb in the same spectral region (which otherwise triggers comparable thermal effects), thus making this approach very appealing for the design of hidden anti-counterfeiting methods.

As an additional proof of concept, to show the generality and versatility of our approach, we also inkjet-printed the same ink dispersion directly on a piece of paper: Figure S15 shows how the printed figures appear only through the thermal camera under NIR irradiation remaining invisible to the naked eye. Moreover, it is worth highlighting that the printed samples can be thermally visible also with simple direct exposure to sunlight, as tested with the reference square samples printed on PEN (also in Figure S15).

As we introduced earlier, the light scattering of the NPs makes the printed figures slightly distinguishable under certain angles. To avoid this problem and realize truly “invisible” prints, we investigated the use of a coating for reducing the contrast created by the different light scattering properties of the printed ITO NPs and the PEN surface. To do so, we coated a printed sample with a thin layer of parylene C. We chose this polymer for its ability to form smooth films that are characterized by reduced optical scattering, its high transmittance in the visible spectrum, and the possibility to form uniform and controlled depositions from gas phase.^60^ As displayed in Figure 5c, the scattering is effectively reduced due to the lower refractive index difference between the NPs and their surroundings. We found that covering the prints with a thin layer of parylene (3 μm in the specific case, see the SEM image in Figure 5d) can strongly reduce the contrast, making the printed item substantially invisible. Notably, the presence of parylene layer seems to slightly enhance the thermal effect on the printed sample (see Figure S16), probably due to the low thermal conductivity of parylene itself, acting as an insulating layer with respect to conductive and convective cooling.

## 3 Conclusions

In conclusion, in this study, we propose a thermoplasmonic active invisible ink for anti-counterfeiting applications, based on a tin-doped indium oxide nanoparticle (ITO NP) colloidal dispersion, presenting plasmonic resonance in the near infrared while being transparent in the visible range. The functional ink can be cast by inkjet printing to create nanometric transparent films with different motifs directly on the target transparent substrate, opening the way to scalable low-cost production of invisible, photothermally active labels. We verified the thermoplasmonic effect of the samples made by printing ITO NPs on the PEN substrate by irradiating them with a NIR lamp and observing the dynamic temperature mapping from the thermal images acquired with an IR thermal camera. After the assessment and optimization of the printing process, we built a demonstrator showing the applicability of this anti-counterfeiting method. We printed a QR code that was invisible to the naked eye while clearly visible in thermal images under NIR radiation. This new procedure can be effective in fabrication and decryption of security labels against counterfeiting as the ink is truly transparent and the thermal reading can be very fast (figures appear/disappear in less than 1 s). Moreover, the fabrication method is difficult to be replicated by counterfeiters. We believe that this approach will contribute to the improvement of anti-counterfeiting systems, also in combination with complementary technologies.

## 4 Material and Methods

### 4.1 Materials

All samples were prepared under a nitrogen atmosphere using commercially available reagents. Tin(VI) acetylacetonate dichloride (≥99%), oleylamine (≥70%), oleic acid (90%), 1-octadecene (ODE) (90%), hexane (≥99%), toluene (≥99.7%), and ethanol (99.9%) were purchased from Aldrich Chemical Co. Indium(III) acetylacetonate (98%) was from Strem Chemicals Co.

### 4.2 Synthesis of ITO NPs

2.4 mmol of In(acac)_3_ and Sn(acac)_2_Cl_2_ were dissolved in 20 mL of octadecene in the presence of oleylamine (6 mmol) and oleic acid (6 mmol). The Sn-to-In ratio was tuned to reach the desired doping level (from 0 to 15%). Under constant stirring and under vacuum, the reaction mixture was brought to 80 °C and maintained for 30 min, after which the temperature was increased up to 160 °C under a nitrogen atmosphere and maintained for 1 h. The temperature was then raised up to 310 °C and maintained for 2 h. After the synthesis, the reaction mixture was cooled down to room temperature and 1 mL of oleic acid was added under stirring. The NPs were washed twice through centrifugation in ethanol for 5 min at 4400*g* and dispersed in toluene or hexane for further characterization. The samples were labeled according to the doping percentage (ITO-0 for 0% doping, ITO-2.5 for 2.5% doping, etc.).

### 4.3 Characterization of ITO NPs

#### 4.3.1 Morphological and Structural Characterization

The average size, size distribution, and shape of ITO NPs were determined by transmission electron microscopy (TEM) using a JEOL 100 SX, operating at 100 kV. Samples were prepared by drop drying a diluted suspension of NPs in hexane onto 200 mesh carbon-coated copper grids. The recorded TEM images were analyzed with the ImageJ software,^61^ and the mean diameter and the size distribution were obtained by statistical analysis over 300 particles.

The mean crystallite diameters, DXRD, and lattice parameters, *a*, were evaluated by powder XRD measurements using a Bruker D8 Advance diffractometer equipped with a Cu Kα radiation and operating in a θ–θ Bragg Brentano geometry at 40 kV and 40 mA. The evaluation of XRD patterns was performed with TOPAS software (Bruker) using the method of the fundamental parameter approach considering the cubic space group *Ia*3. The reference pattern of In_2_O_3_ (powder diffraction file, PDF-06-0416) was plotted as a reference in the main test.

The Sn doping level of the different samples was determined by ICP-AES measurements, performed in triplicate by a Varian 720-ES inductively coupled plasma atomic emission spectrometer. Dried solid samples (∼1 mg) were digested in concentrated aqua regia (HCl suprapure and HNO_3_ sub-boiled in 3:1 ratio) and in the presence of H_2_O_2_, diluted with ultrapure water (≥18 MΩ/cm) and analyzed using Ge as the internal standard. Calibration standards were prepared by gravimetric serial dilution from a monostandard at 1000 mg/L. The wavelengths used for In and Sn were 325.6 and 189.9 nm, respectively. The dopant content is defined as , where *n*_Sn_ and *n*_In_ are the moles of substitutional dopant (Sn) and lattice cation (In), respectively. Thermogravimetric analysis was carried out using a Mettler TGA Q500 instrument on ∼1 mg of dried sample, placed in an alumina melting pan. All samples were analyzed in the temperature range from 25 to 600 °C with a scan rate of 10 °C/min under nitrogen flux. The inorganic content of the NPs was determined from the residual weight fraction at 600 °C and the surfactant coating was quantified from the weight loss.

#### 4.3.2 Optical Characterization

Extinction spectra were collected using a commercial Cary 5000 UV–Vis–NIR spectrophotometer (Agilent), which can work in the spectral range 200–3300 nm. The spectra were collected in 1 cm quartz QX cuvettes. C_2_Cl_4_ was used as the solvent for its transparency in the spectral region of interest. The concentration of the samples was chosen to have an optical density in the range 0.5–1.5. The extinction coefficient of ITO-2.5, ITO-5, and ITO-10 was evaluated from the extinction spectra of ITO NP dispersions at known concentration, according to the following relation, in agreement with the procedure reported by Staller *et al.*:^56^
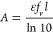
where *A* is the extinction measured in a base 10 log scale at the maximum of the plasmonic peak; *l* is the optical path (1 cm in our case); ε is the extinction coefficient (expressed in μm^–1^), *f_v_* is the volume fraction calculated from the elemental composition obtained through ICP-AES analysis, assuming an elemental composition (In + Sn)_2_O_3_ and a density of 7140 mg/mL; ln 10 is used to convert extinction to the natural log scale.

### 4.4 ITO NP Ink Formulation and Printing Characterization

#### 4.4.1 Inkjet Printing of ITO NPs

A hexane dispersion of NPs with 10% doping (ITO-10) obtained after the washing procedure was centrifuged and re-dispersed in 1,2-dichlorobenzene at a nominal concentration of 36 mg/mL. These dispersions were subsequently used for inkjet printing on transparent substrates. The same procedure has been followed for preparing non-plasmonic reference ink, starting from undoped ITO NPs (sample ITO-0). The chosen printing substrate consisted of a 25 μm-thick PEN-film TEONEX purchased from Pütz-Folien (Taunusstein, Germany), which allows both flexibility and transparency. Inkjet printing was performed with a Dimatix Materials Printer DMP- 2800 (Fujifilm Corp., Japan) by using Dimatix disposable cartridges with a 10 pL nozzle volume (DMC-11610). The ITO NP dispersion acting as ink was loaded into the cartridge. Before each printing step, 60 s of O_2_ plasma at a pressure of 0.6 mbar (100 W) treatment was carried out on the PEN substrates by using a Plasma Cleaner System (Gambetti, Italy) in order to increase the wettability of the target substrate. All the printing processes were carried out in a clean room facility at room temperature.

Different samples have been prepared for specific characterization experiments. In particular, (i) for printing parameter characterization (thermal behavior and optical transmittance), squares of 1 cm^2^ have been printed on the same substrate sample, by varying parameters, i.e., by using drop spacings (DS) of 25 and 50 μm (corresponding to resolutions of 1016 and 508 dpi), with 1 and 2 printed layers; the samples are consequently named 25DS 1 L, 50DS 1 L, 25DS 2 L, and 50DS 2 L; (ii) for the profilometry measurements (to evaluate deposited ink thickness), 250 μm-wide 1 cm-long lines have been printed on a silicon wafer by using drop spacings (DS) of 25 and 50 μm with 1 and 2 printed layers; (iii) all other patterns presented in this study have been printed as a single layer with a 25 μm DS (25DS 1 L).

To evaluate maximum spatial resolution achievable by thermal imaging, two different designs have been used: the first consisted of four straight lines with the same length of 2 cm and widths of 2500, 1000, 500, and 250 μm, separated from each other by a 5 mm distance. The second one consisted of 5 straight lines with the same length of 2 cm and thickness of 2500 μm, separated from each other by distances of 2500, 1000, 500, and 250 μm, respectively. The samples have been used to evaluate the minimum detectable thickness and minimum line separation.

#### 4.4.2 Characterization of the Printed Samples

Thermal characterization of ink-jet printed paths on PEN has been performed by irradiating the samples with an infrared lamp of 100 W nominal power from Philips placed about 20 or 50 cm above it, depending on the experiment, as specified in the main text. Thermal imaging has been performed using a FLIR A300 camera (FLIR) and related ResearchIR 4 software (used for the analysis and post-processing). The thermal camera was placed at an about 20 or 50 cm distance from the sample for the measurement.

The UV–Vis transmittance spectra of the printed square samples were measured directly on the printed samples 25DS 1 L, 50D S 1 L, 25D S 2 L, and 50D S 2 L and on bare PEN by using a LAMBDA 650 spectrophotometer (PerkinElmer). The IR absorbance spectra of the 25DS 1 L square sample was measured as well by using a Cary 5000 UV–Vis–NIR spectrophotometer (Agilent) in the 800–3000 nm spectral range.

Thickness measurements were carried out with a P6 stylus profilometer (KLA-Tencor) on purposely prepared samples.

Optical microscopic images have been acquired using a DCM 3D confocal profilometer (Leika) at 10× magnification with the multiple images stitching option.

Scanning electron microscopy images of the printed ITO NPs on PEN and focused ion beam-milled cross sections of Parylene-coated samples were obtained with a Dual Beam FIB/SEM Helios Nano-Lab 600i (FEI). Scanning electron microscopy images of the cross sections were obtained under a sample tilt angle of 52° (accelerating voltage 10 kV).

To design the QR code demonstrator, a free online QR code generator was used linking it to a custom web page (free QR code generator: https://www.websiteplanet.com/webtools/free-qr-code-generator/).

The Parylene Coater PDS2010 (Specialty Coating Systems) was used to deposit a layer of parylene (2 g of sublimated parylene, corresponding to 3 μm of nominal deposited thickness) on the selected samples to qualitatively evaluate the visibility decrease of the ITO NP pattern in reflection conditions.

### 4.5 Thermal Simulations

COMSOL Multiphysics v5.6 was used for the implementation of the finite element model and simulations. A time-dependent study using the Heat Transfer in Solids module was considered. The PEN substrate was modeled with dimensions of 200 × 200 × 0.025 mm^3^ and, on top of this, at its center, a 10 × 10 mm^2^ ITO NPs square was placed (simulated thickness 66 nm, as measured from profilometry on 25 um DS 1 layer square). All the material properties are reported in Figure S12. Based on the experimental results, both the initial temperature value for the whole model and the ambient temperature were set at 26.2 °C. In order to consider the coupling of heat conduction and radiation, the Stefan–Boltzmann law was added to the model as a boundary condition so as to consider heat radiation exchange between all the surfaces of the geometry and the ambient environment. Both the PEN and ITO NP dispersions were modeled as black bodies – emissivity was assumed independent of temperature and fixed at a constant value of 0.95. The heating element – i.e., the NIR lamp in the experiments – was modeled by applying a heat flux directly on the ITO NP square for which the heat rate was specified in terms of a constant power per surface area. In particular, the applied power was estimated by the measured absorbance of the printed sample and the theoretical emission spectra of the IR lamp and it was set equal to 54.1 W/m^2^ (details in the Supporting Information – Power Flux Estimation). Since the geometry was considered to be entirely in the air, the heat convention phenomena between the geometry and the ambient were implemented by using a heat flux as a boundary condition on all the surfaces. The heat transfer coefficient of air was set at 2.5 W/(m^2^ K).^62^ A free triangular mesh was generated on the *xy* plane with the element size in the range of 0.5 × 10^–6^ to 5 × 10^–4^ m for the ITO square and 7.0 × 10^–4^ to 0.011 m for the PEN square. The built mesh has been swept with a 5-point fixed distribution for the ITO geometry and a 20-point fixed distribution for the rest of the PEN substrates. The time-dependent study was performed from 0 up to 20 s using the default solver settings except for the relative tolerance, which was reduced to 10^–3^. A parametric sweep was performed on the parameter representing the thickness of the substrate in the range 10–1000 μm. Further details on thermal simulations can be found in the Supporting Information.

## Supporting Information Available

Figures S1–S16: Additional experimental details, materials, and methods, including absorption spectra modeling and fitting, and thermal simulations (PDF)
Video S1: Demonstrative thermo QR code detection in real time (MP4)
