# Supplementary material for: Invisible Thermoplasmonic Indium Tin Oxide Nanoparticle Ink for Anti-counterfeiting Applications
Source: ACS Appl Mater Interfaces. 2022 Jul 22;14(30):35276–86. doi: 10.1021/acsami.2c10864 (PMC9354021; doi:10.1021/acsami.2c10864)
Supplement: Supplementary file 1 — am2c10864_si_001.pdf [file am2c10864_si_001.pdf]

Supporting Information

Invisible thermoplasmonic ITO nanoparticles ink for anti-counterfeiting applications

Arianna Mazzotta†,[a,b], Alessio Gabbani†,[c], Marco Carlotti†,[a], Marina Ruggeri[c], Elvira Fantechi[c], Andrea Ottomaniello[a], Francesco Pineider*,[c], Andrea Pucci*,[c], Virgilio Mattoli*,[a]

[a] Center for Materials Interfaces, Istituto Italiano di Tecnologia, Viale R. Piaggio 34, Pontedera 56025, Italy

[b] The Biorobotics Institute, Scuola Superiore Sant'Anna, Viale R. Piaggio 34, Pontedera 56025, Italy

[c] Department of Chemistry and Industrial Chemistry, University of Pisa, Via Moruzzi 13, 56124 Pisa, Italy

* Corresponding authors: francesco.pineider@unipi.it; andrea.pucci@unipi.it; virgilio.mattoli@iit.it

† These authors contributed equally

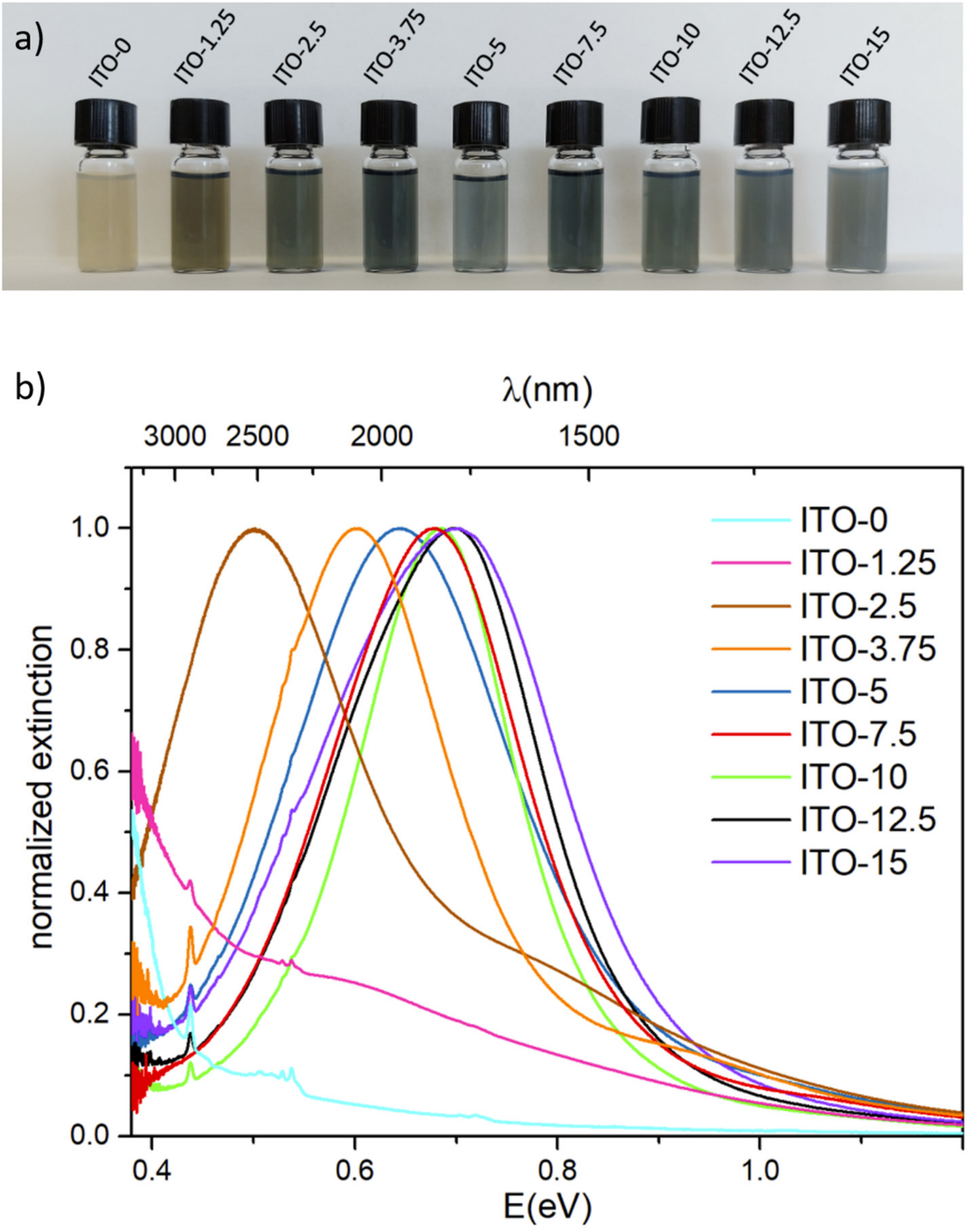

Figure S1 –Picture (a) of the synthesized samples at high concentration (36 mg/mL) in hexane dispersions and normalized extinction spectra (b) of diluted nanoparticle dispersions in C_2Cl_4 at various Sn doping levels. ITO-x sample is related to x% of Sn doping of pure In_2O_3, i.e. ITO-0 (Sn 0%), ITO-1.25 (Sn 1.25%), ITO-2.5 (Sn 2.5%), ITO-3.75 (Sn 3.75%), ITO-5 (Sn 5%), ITO-10 (Sn 10%), ITO-12.5 (Sn 12.5%) and ITO-15 (Sn 15%).

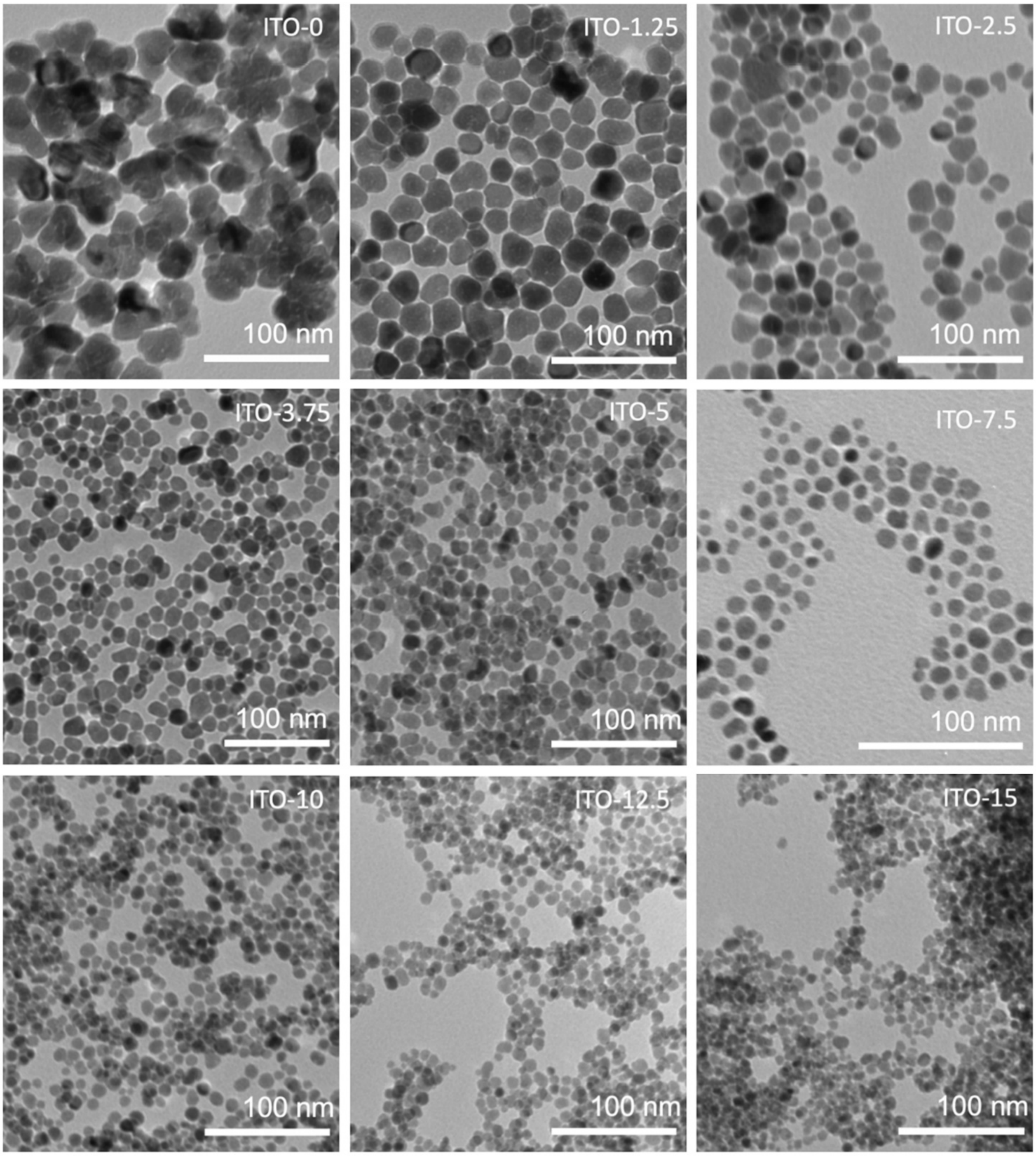

Figure S2 – Representative TEM images of samples ITO-0 (Sn 0%), ITO-1.25 (Sn 1.25%), ITO-2.5 (Sn 2.5%), ITO-3.75 (Sn 3.75%), ITO-5 (Sn 5%), ITO-10 (Sn 10%), ITO-12.5 (Sn 12.5%) and ITO-15 (Sn 15%).

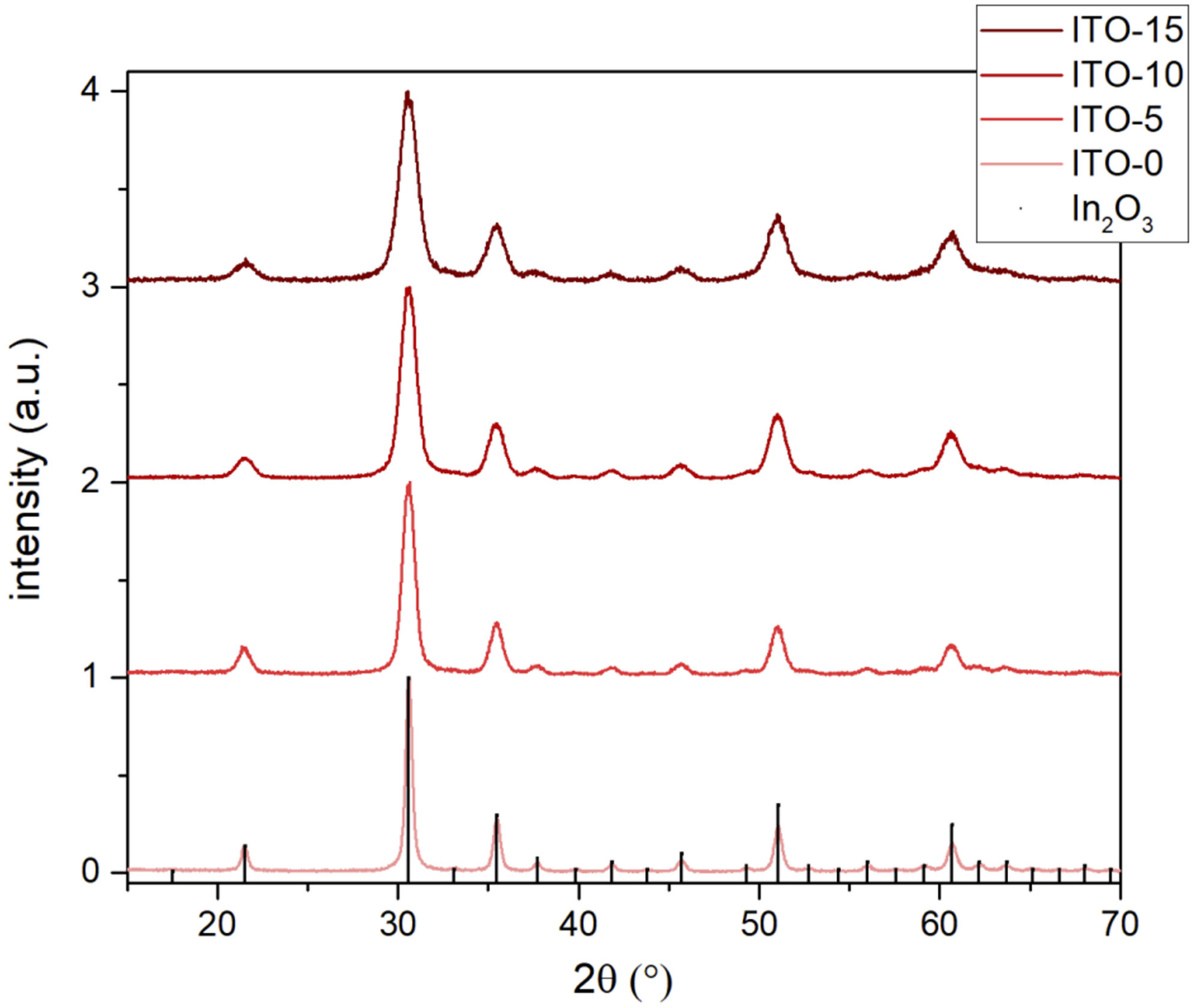

Figure S3 – XRPD diffraction pattern of representative samples ITO-0, ITO-5, ITO-10 and ITO-15 together with the reference pattern of In_2O_3 (Powder Diffraction File, PDF-06-0416).

Table S1 – Main features of nanoparticle dispersions prepared at various Sn doping levels. a) Sn% set in the reaction mixture from the initial ratio of metal precursors; b) Sn% from ICP-AES measurements; c) average diameter and standard deviation obtained from size distribution analysis of TEM images; d) mean crystallite size (errors are given in brackets) of nanophase materials obtained from the fit of XRD diffractograms and using Scherrer equation;[1] e) lattice parameter (errors are given in brackets) obtained from the XRD diffractograms; f) the optical band gap obtained from the extinction spectrum using the TauC plot.[2]

Sample	Initial Sn%[a]	Final Sn%[b]	Diameter (nm)[c]	d_{XRD}(nm)[d]	a(Å)[e]	Band Gap (eV)[f]
ITO-0	0	0	21 ± 4	20.6(1)	10.1182(2)	3.62
ITO-1.25	1.25	1.0	19 ± 3	16.86(8)	10.1231(3)	3.60
ITO-2.5	2.5	2.7	15 ± 3	14.32(6)	10.1259(3)	3.76
ITO-3.75	3.75	3.9	12 ± 3	12.11(7)	10.1277(5)	3.75
ITO-5	5	5.1	11 ± 2	10.79(6)	101235(5)	3.76
ITO-7.5	7.5	7.2	10 ± 2	9.31(4)	10.1372(3)	3.77
ITO-10	10	10.2	9 ± 2	8.63(5)	10.1245(5)	3.76
ITO-12.5	12.5	12.8	9 ± 2	7.96(9)	10.1306(8)	3.73
ITO-15	15	15.8	7 ± 1	7.26(9)	10.1271(8)	3.76

[1] C. F. Holder and R. E. Schaak, ACS Nano, 2019, 13, 7359–7365
[2] J. Tauc, Materials Research Bulletin, 1968, 3, 37–46

Table S2 –Parameters of normalized extinction spectra of nanoparticle dispersions, prepared at various Sn doping levels. The peak positions and peak width were extracted through a fitting with pseudovoigt peak functions[3]. In the case of NPs with doping below 7% a second peak function is needed to obtain a good fitting. This can be rationalized with the presence of a second plasmonic resonance due to the increased shape anisotropy of the particles detected by TEM[4]. However, the entity of this second contribution is significant only for the 2.5% NPs. a) Sn% from ICP-AES measurements; b) peak area normalised for the total area; c) peak center (in eV); d) peak width (in eV).

Sample	Sn%[a]	A_1/A_{tot}[b]	γ_1(eV)[c]	E_1(eV)[d]	A_2/A_{tot}[b]	γ_2(eV)[c]	E_2(eV)[d]
ITO-2.5	2.7	0.58	0.21	0.50	0.42	0.40	0.74
ITO-3.75	3.9	0.89	0.22	0.60	0.11	0.30	0.91
ITO-5	5.1	0.93	0.26	0.64	0.07	0.35	0.95
ITO-7.5	7.2	0.99	0.22	0.67	0.01	0.11	1.06
ITO-10	10.2	1	0.19	0.68	-	-	-
ITO-12.5	12.8	1	0.25	0.68	-	-	-
ITO-15	15.8	1	0.29	0.68	-	-	-

[3] A. Gabbani *et al.* ACS Appl. Nano Mater. 2021, 4, 1057–1066
[4] Y. Gu, Z. Zhu, J. Song and H. Zeng, Nanoscale 2017, 9, 19374-19383

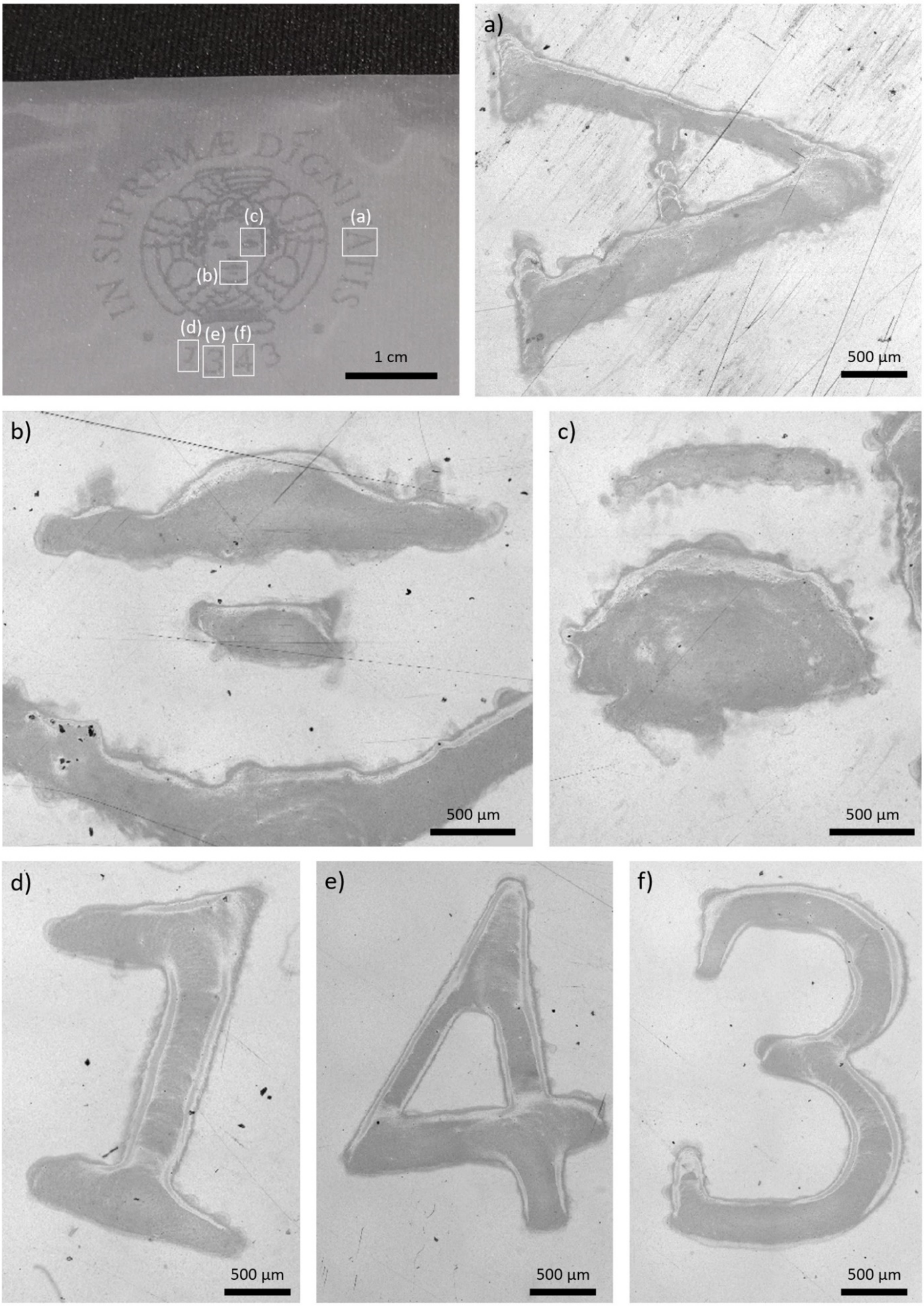

Figure S4 – Optical microscopy images of "cherubin" ITO NPs printed sample (top left), showing high printing quality in selected details (a-f). Polarization contrast provided by the microscope makes clearly visible the printed details, otherwise invisible at naked eyes.

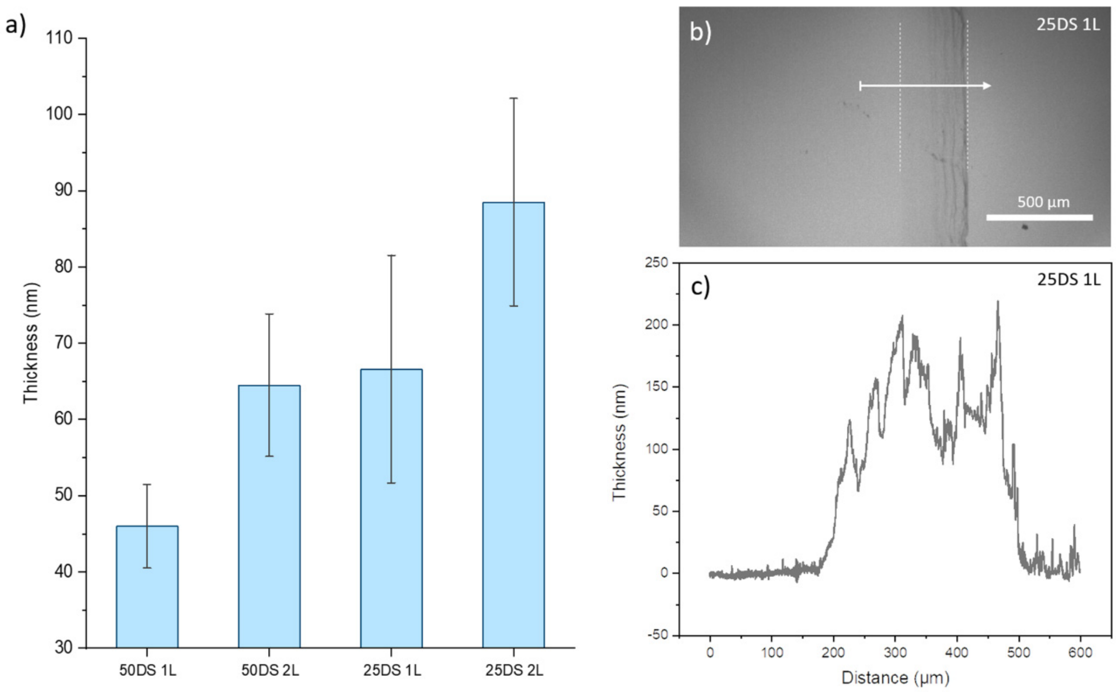

Figure S5 – Results of profilometry of 250 µm-width printed lines: histogram of thickness at different printing parameters (a) with a representative thickness profile of sample 25DS 1L (c) and related microscopic picture (b). Error bars are confidence intervals (α= 0.05).

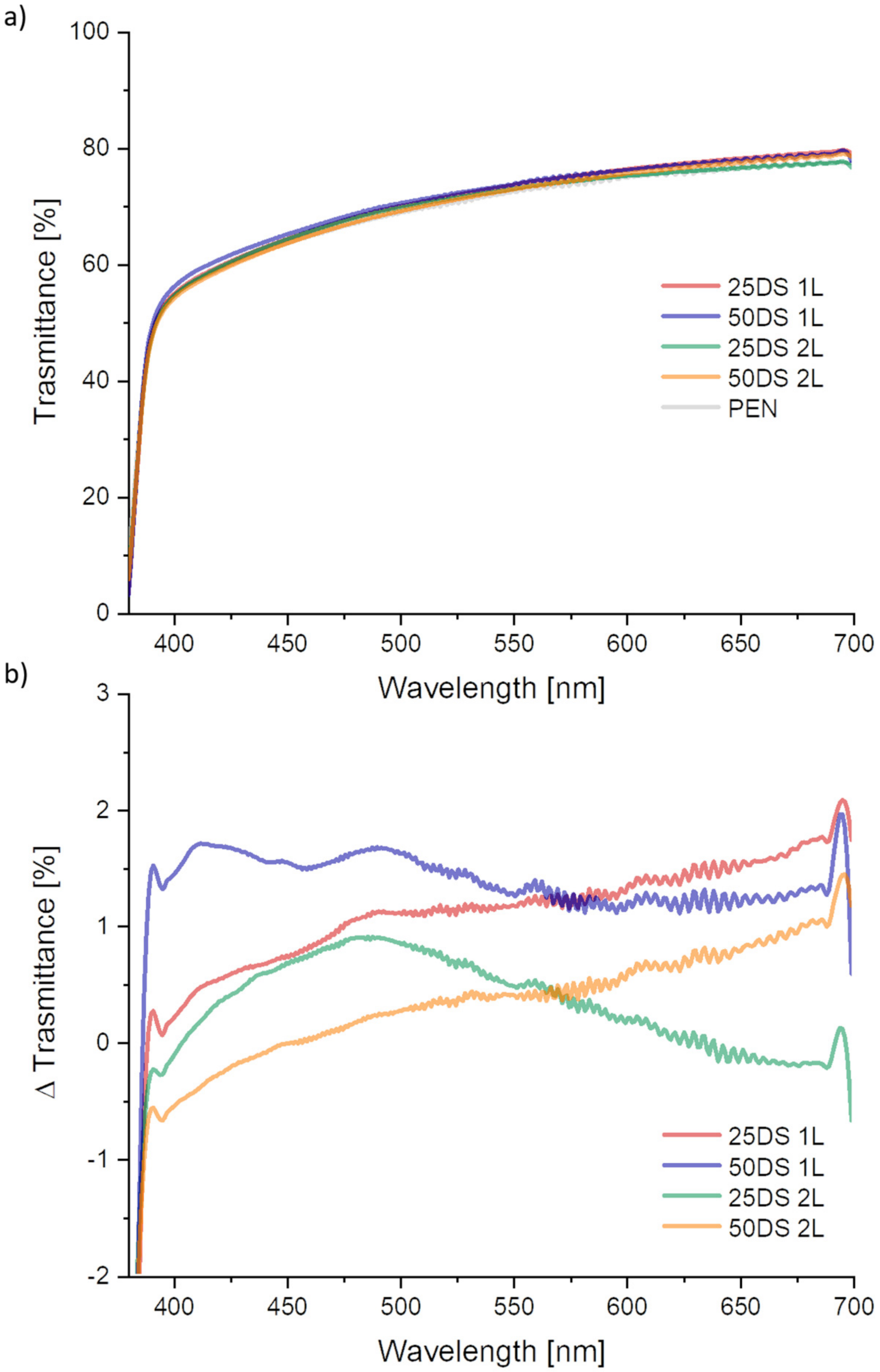

Figure S6 – a) UV-Vis transmittance of different samples of ITO ink printed on PEN with different printing parameters. It is noteworthy that the absorbance of the simple PEN and the printed samples (at all the concentrations) is mostly identical, indicating a very low contribution of printed ITO ink to the transmittance decrease. b) Difference between the latter transmission spectra of the printed samples and the PEN substrate. The average decrease of transmittance due to the absorption of ITO NPs is in the order of 1-1.5%, thus the printed ink can be considered highly transparent.

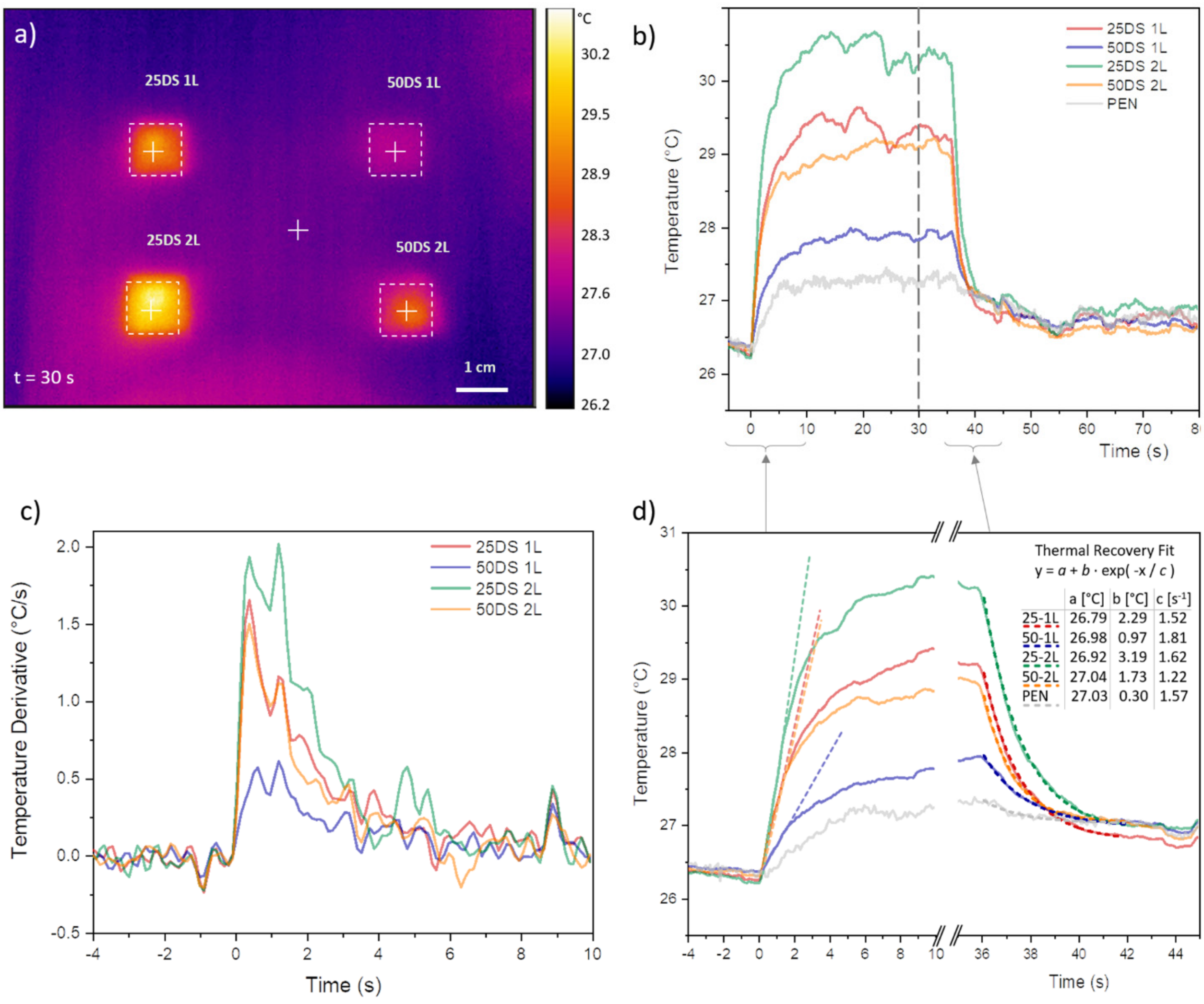

Figure S7 –Thermal dynamic of ITO-10 NP square samples printed with different parameters on the same substrate (as in Figure 3 of main text), exposed to IR lamp irradiation (100W@50 cm); sample 25D 1L, 25D 2L, 25D 2L and 25D 2L refers to various combinations of drop spacing (DS = 25, 50 µm) and number of printed layers (L = 1, 2). a) Thermal images at regime. b) Plot of temperature dynamics of the same samples, averaged with a ROI (region of Interest) of 0.5x0.5cm^2 placed in the center of the printed squares (as in (a)). PEN substrate has been acquired at the center of the thermal images (as in (a)). c) Derivative of the temperature vs. time for the different samples. d) detailed view of the temperature plot, with zoom on starting heating (left) and on temperature recovery after turning-off the heating source (right). For starting heating, the initial derivative value (dotted lines) are superimposed to experimental curves for comparison. Exponential decay fittings are superimposed to the thermal recovery curves; time constant is similar for all the curves (within 1.5 s ± 0.3 s) indicating the printed layer does not affect significantly the cooling phase, due to the extreme low thickness of ITO film corresponding to a small absolute thermal capacity.

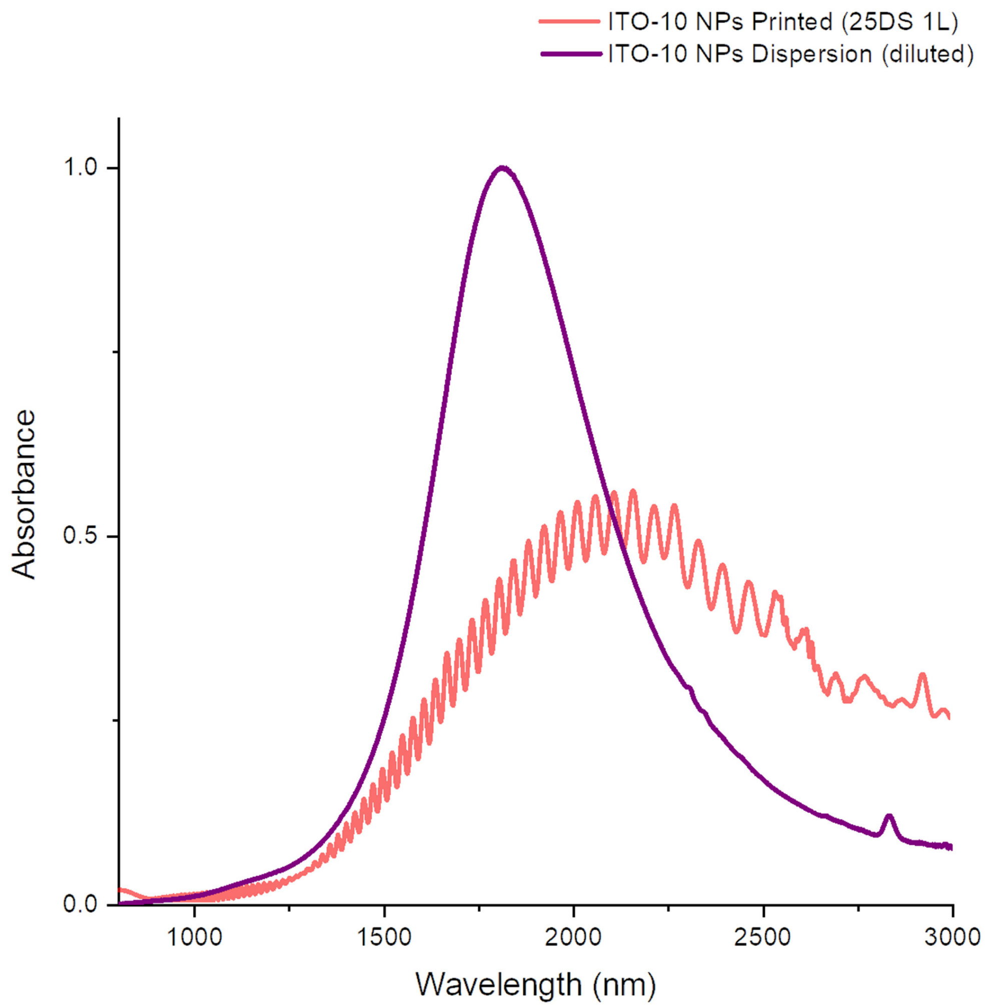

Figure S8 – Absorbance of printed ITO nanoparticles and diluted dispersion. It is worth to notice that in the printed sample the peak is shifted toward longer wavelengths while the full width at half maximum (FWHM) increases. This is mainly due to the increase of the refractive index of the sample, because of to the larger NPs volume fraction, as explained in the following section. In the printed ITO sample spectrum the absorbance of PEN substrate has been subtracted.

Modelling and Fitting of ITO nanoparticles dispersions and solid film

Mie theory. Mie theory considers isolated spherical NPs immersed in a dielectric medium. Interaction between NPs is not considered, as well as changes in the dielectric constant of the medium due to the presence of neighbouring NPs.

With this model, when the NPs are much smaller than the incoming wavelength (D << λ), the extinction cross section can be significantly simplified according to equation (S1), neglecting multipolar modes and scattering contribution. This regime is called the quasi-static approximation. In our studies ITO NPs are smaller than 20 nm, thus allowing us to use the quasi-static approximation.[5]

$$\sigma_{ext} = \sigma_{abs} = k\sqrt{\varepsilon_m} Im[\alpha] = \frac{E}{\hbar c}\sqrt{\varepsilon_m} Im[\alpha] = \frac{E}{\hbar c}\sqrt{\varepsilon_m}\frac{\pi D^3}{2} Im\left[\frac{\varepsilon_{NP}(E)-\varepsilon_m}{\varepsilon_{NP}(E)+2\varepsilon_m}\right] \qquad \text{(S1)}$$

where α is the polarizability for a spherical NP, k is the wavevector, E is the photon energy, $\hbar$ is the barred Plank constant, and c the light velocity, while ε_{NP} and ε_m are the dielectric function of the NP and the surrounding medium respectively. ε_{NP} is a frequency dependent and complex function, while ε_m is generally constant and real for a medium which is transparent in the spectral range of interest. In this regime, the NP size is just a scaling factor in equation (S1), but it does not modify the lineshape and position of the plasmonic response.

ε_{NP} can be expressed as the sum of all the contributions related to the optical transition occurring in the system. For a plasmonic system, the main contribution (at wavelengths close to the plasmonic resonance) is generally the one of free electrons and it is generally expressed analytically using the *Drude formulation*.[6] If other optical transitions are present in the spectral range of interest, i.e. inter-band transition in noble metals, this contribution should be considered.[7] For Au and Ag generally the bulk experimentally determined dielectric function is inserted into equation (S1) to calculate the optical response.

For ITO, interband (bandgap) transitions occur in the UV range, which is far from the plasmonic resonance. Drude formulation (equation S2) is thus commonly used for ITO and other plasmonic doped semiconductors with wide band gap. In this formulation, the contribution to ε of the bulk material is generally inserted as an additive term, ε_∞ which is a real quantity. This contribution accounts for the background polarizability of the material. For ITO, values of ε_∞ = 4 are generally reported in the literature.[8]

---

[5] Kreibig, U.; Vollmer, M. Optical Properties of Metal Clusters; Toennies, J. P., Gonser, U., Osgood, R. M., Panish, M. B., Sakaki, H., Lotsch, H. K. V., Series Eds.; Springer Series in Materials Science; Springer Berlin Heidelberg: Berlin, Heidelberg, 1995; Vol. 25.

[6] Ashcroft, N. W.; Mermin, N. D. Solid State Physics; Holt, Rinehart and Winston: New York, 1976.

[7] Johnson, P. B.; Christy, R.-W. Optical Constants of the Noble Metals. Physical review B 1972, 6 (12), 4370 | Etchegoin, P. G.; Le Ru, E. C.; Meyer, M. An Analytic Model for the Optical Properties of Gold. The Journal of Chemical Physics 2006, 125 (16), 164705. | Kreibig, U.; Zacharias, P. Surface Plasma Resonances in Small Spherical Silver and Gold Particles. Z. Physik 1970, 231 (2), 128–143.

[8] Mendelsberg, R. J.; Garcia, G.; Li, H.; Manna, L.; Milliron, D. J. Understanding the Plasmon Resonance in Ensembles of Degenerately Doped Semiconductor Nanocrystals. J. Phys. Chem. C 2012, 116 (22), 12226–12231. | Ederth, J.; Johnsson, P.; Niklasson, G. A.; Hoel, A.; Hultåker, A.; Heszler, P.; Granqvist, C. G.; van Doorn, A. R.; Jongerius, M. J.; Burgard, D. Electrical and Optical Properties of Thin Films Consisting of Tin-Doped Indium Oxide Nanoparticles. Phys. Rev. B 2003, 68 (15), 155410.

$$\varepsilon_{NP}(\boldsymbol{E}) = \varepsilon_\infty - i\frac{\hbar^2 e^2 \boldsymbol{N}}{\boldsymbol{m}^* \varepsilon_0 \boldsymbol{E}}\frac{\boldsymbol{\gamma} - i\boldsymbol{E}}{E^2 + \boldsymbol{\gamma}^2} \quad \text{(S2)}$$

In equation (S2), free electron parameters are introduced: **N** is the free electron density, γ is the damping parameter, which accounts for electron scattering and is related to the full-width ad half maximum (FWHM) of the plasmonic peak, **m*** is the electron effective mass, while the other parameters are constant (electron charge *e*, dielectric permittivity of vacuum ε_0, and plank constant $\hbar$).

For ITO, generally the damping constant is considered as frequency-dependent, and an empirical function is used:[9]

$$\gamma(\omega) = \gamma_L - \frac{\gamma_L - \gamma_H}{\pi}\left[tan^{-1}\left(\frac{\omega - \gamma_x}{\gamma_w}\right) + \frac{\pi}{2}\right] \quad \text{(S3)}$$

where a low frequency and a high frequency regime are defined, with damping constant γ_L and γ_H respectively. γ_x is the threshold between the two regimes, while γ_w is the width of the transition between the two regimes.

Drude dielectric function (equation S2) is then inserted into equation (S1) to obtain the extinction cross section of the NPs. From the extinction cross section one can calculate the absorbance of a solution or a film containing the NPs:[8]

$$A_{Mie} = \frac{NL\sigma_{abs}}{\ln(10)} = \frac{3f_v L\sigma_{abs}}{\ln(10)4\pi R^3}, \quad \text{(S4)}$$

where *N* is the particle number density, f_v is the volume fraction of the NPs in the medium, *L* is the optical path (cuvette length in the case of a solution or film thickness in the case of NPs embedded in a polymer matrix), *R* is the NPs radius, and σ_{abs} the NPs absorption cross section.

This approach to calculate the optical spectrum is valid only for diluted samples, i.e. for volume fractions below 0.1. The model is thus suitable for diluted solutions, such as ITO NPs dispersed in C_2Cl_4.

However, when NPs concentration increases, we may have two concurring effects that modify the optical response with respect to the one predicted by Mie theory:

1. At high volume fraction the dielectric constant of the medium that surrounds each NP is different than the one of the bare matrix, as the NPs contribution start to be significant. For ITO for instance, the real part of ε is higher than the solvent (2.26 for C_2Cl_4). This is expected to red shift the plasmonic peak, and it is not predicted by Mie theory.
2. When the NPs volume fraction is very high, the NPs are very close and they can interact. In this case, due to dipolar interactions between NPs, the polarizability of each NP is affected by the neighbouring ones. In this case generally inter-particle interactions must be taken into account to calculate the plasmonic resonance of densely packed films or NPs embedded in a polymeric matrix at high concentration. Moreover, depending on the preparation

[9] Hamberg, I.; Granqvist, C. G. Evaporated Sn-doped In2O3 Films: Basic Optical Properties and Applications to Energy-efficient Windows. Journal of Applied Physics 1986, 60 (11), R123–R160. | Hamberg, I.; Granqvist, C. G. Optical Properties of Transparent and Heat-reflecting Indium Tin Oxide Films: The Role of Ionized Impurity Scattering. Appl. Phys. Lett. 1984, 44 (8), 721–723.

technique, during the formation of polymeric films containing the NPs, aggregates of closely packed NPs may form.

To take into account the first effect, Maxwell-Garnet effective medium approach can be used.

Maxwell-Garnet effective medium approach (MG). In this approach, an effective dielectric function ε_{eff} is considered for the sample, in which spherical inclusions with volume fraction f_v and dielectric function ε_{NP} are dispersed in a host matrix (i.e. a polymer or a solvent) with dielectric constant ε_m. The full dielectric function of the materials (made of the NPs and the matrix) is thus expressed according to equation (S5), in which the dielectric function of matrix and NPs are "mixed" according to Maxwell-Garnet mixing rules:[10]

$$\varepsilon_{eff} = \frac{(1+2f_v)\varepsilon_{NP}(\omega)+2(1-f_v)\varepsilon_m}{(1-f_v)\varepsilon_{NP}(\omega)+(2+f_v)\varepsilon_m}\,\varepsilon_m \tag{S5}$$

The absorbance of the sample can then be calculated from the effective dielectric function according to equation (6): 11

$$A_{MG} = \frac{L(cm)}{ln10} 7.16 \cdot 10^4 \cdot E \cdot \left(-\varepsilon_{eff,1} + \sqrt{\varepsilon_{eff,1}^2 + \varepsilon_{eff,2}^2}\right)^{\frac{1}{2}} \tag{S6}$$

$\varepsilon_{eff,1}$ and $\varepsilon_{eff,2}$ are the real and imaginary part of ε_{eff}, while L is the optical path in cm, and E the photon energy. The units of the constant $7.16 \cdot 10^4$ are cm^{-1}, and the constant originates from this term: $4\pi/(hc\sqrt{2})$. The derivation of equation (6) can be found in Fox's book,[11] when the relation between imaginary part of refractive index of a material, and the optical density is expressed. Indeed, optical density is proportional to the optical cross section of the material which is proportional to the imaginary part of its refractive index. Relation between refractive index and dielectric function are also used to derive equation (S6).

In the work by Mendelsberg *et al.*,[8] a comparison between Mie theory and Maxwell Garnet approach is provided, with a focus on the fitting of ITO and CuSe plasmonic NPs. Mie theory is valid for volume fractions of NPs below 0.01, and is indipendent on the f_v, i.e. the normalized spectra obtained at different volume fractions are superimposable. Maxwell-Garnet (equation S5-S6) should be used for higher volume fraction.

The use of equation (S5-S6) takes into account for the change in refractive index of the sample, due to the increase of the NPs volume fraction.

Fitting of printed ITO NPs. To fit the printed film of ITO NPs, MG approach should be used, as the volume fraction is very high. First, we applied Mie theory (equations S1-S4) on a stable dispersion

[10] Markel, V. A. Introduction to the Maxwell Garnett Approximation: Tutorial. J. Opt. Soc. Am. A 2016, 33 (7), 1244. | Mendoza, D. Composite System of Metallic Gold Particles in Hydrogenated Amorphous Silicon. Journal of Non-Crystalline Solids 1988, 103 (1), 151–153.

[11] Fox, M. Optical Properties of Solids, 2nd ed.; Oxford master series in condensed matter physics; Oxford University Press: Oxford; New York, 2010.

of ITO NPs to determine free electron parameters (namely plasma frequency and damping parameters). These are the fitting performed on ITO-10 dispersed in C_2Cl_4 (see Supporting Figure S10.a), already performed and reported in our previous work, [12] thus obtain the following parameters:

N (x10^{26} m^3)	γ_L (eV)	γ_H (eV)	γ_X (eV)	γ_W (eV)	m*=m/m_e
7.17	0.23	0.085	0.76	0.068	0.27

Instead of expressing m and N separately we can include them in a single quantity, the plasma frequency, which is in our case 1.90 eV.*

Using the same parameters, we calculated Maxwell Garnet Absorbance (A_{MG}) of the NPs in C_2Cl_4 at different volume fractions. In the calculation the product between optical path and volume fraction has been fixed (L x f_v = 2.5 x10^{-6} cm). In this way, the comparison is independent on the number of nanoparticles considered. For Mie theory such calculations give similar absorbance with different volume fractions (not reported). Similar results with respect to the reference paper[8] are obtained. The absorbance is considerably modified by the increase of the volume fraction. In particular, a red shift with the increase in volume fraction is observed, consistently with the increase in the effective permittivity of the medium due to the presence of the ITO NPs. Indeed, the dielectric function that is around each NPs is modified with respect to the matrix when the NP volume fraction is high. This red shift is accompanied by a broadening and a decrease of the intensity of the plasmonic resonance as f_v increase (Supporting Figure S9).

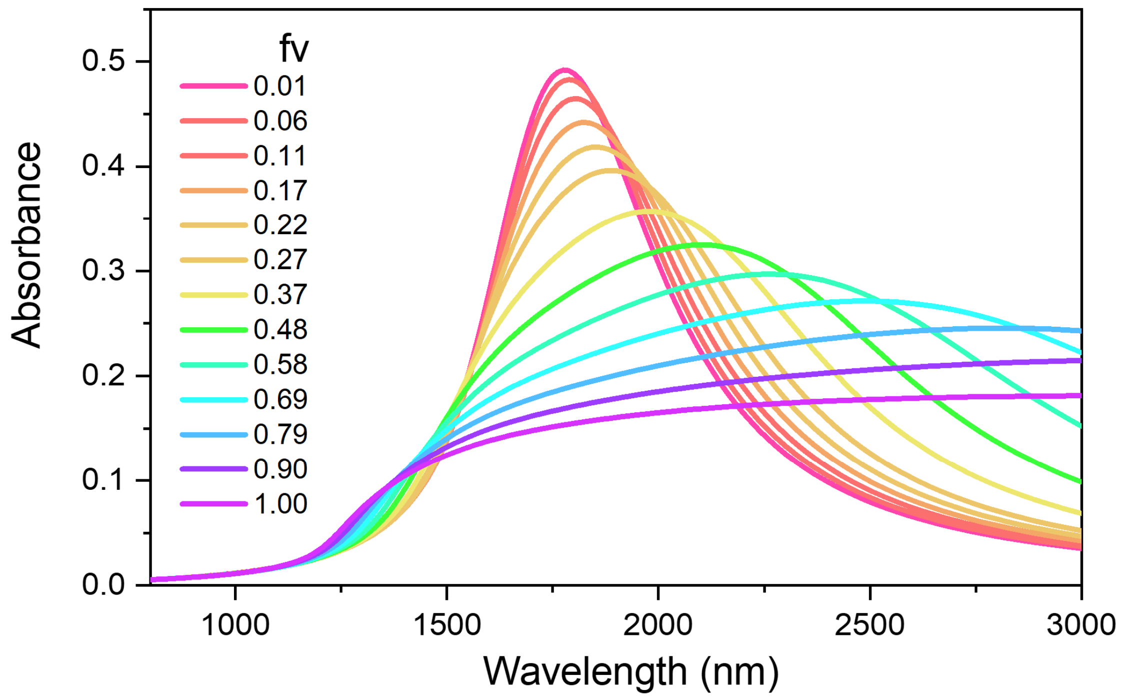

[12] Gabbani, A.; Sangregorio, C.; Tandon, B.; Nag, A.; Gurioli, M.; Pineider, F. Active Magnetoplasmonics with Transparent Conductive Oxide Nanocrystals. arXiv:2104.07772 [cond-mat, physics:physics] 2021.

Figure S9 Absorbance calculated for different volume fraction using Maxwell-Garnet theory, and the Drude parameters obtained from the fitting of the experimental spectrum of ITO-10 NPs dispersed in C_2Cl_4. In the calculation the product between optical path and volume fraction has been fixed (L x f_v = 2.5 x10^{-6} cm).

In the case of our printed ITO film, the volume fraction is expected to be very high as most of the solvent evaporates after inkjet printing of the NPs, leaving on the substrate the ITO NPs surrounded by the oleic acid coating. Since 8% in weight (by TGA analysis) of the NPs is composed by the oleic acid coating (40% in volume), a large part of organic component is expected to be present in the film as coating of the NPs. A comparison between the printed film and the solution is reported in Supporting Figure S8. The spectrum of the printed film is averaged between two measurements in two different points, and the spectrum of the PEN substrate is subtracted. We should point out that the spectrum of the PEN substrate is characterized by the strong interference effects (fine oscillations), which are not completely subtracted as they are modified by the presence of the ITO NPs film. The optical response of the film is red shifted and broadened with respect to the ITO NPs in solution. This can be ascribed to the increase in volume fraction of the ITO NPs.

Due to the high-volume fraction, MG approach (equations S5-S6) was used to fit the spectrum of the printed film. In first approximation we assumed that NPs parameters remain equal to the one of the solution spectra, as the NPs have not been subjected to thermal treatments. Such parameters were fixed during the fitting, while carrier concentration and volume fraction are left free. Optical path is kept equal to the measured film thickness (66 nm). The refractive index of the medium is considered as a weighted average between air (ε = 1) and the organic part which includes the oleic acid coating and part of the 1,2-dichlorobenzene solvent which did not evaporate after the deposition (indeed they have a similar dielectric constant, ε = 2.3). The volume fraction of organic (f_{org}) in the medium that surrounds the NPs is left free during the fitting, while the volume fraction of air in the medium is (1-f_{org}). The fitting obtained is reported in Supporting Figure S10.b. The fitting parameters are reported in the following table.

ω_p (eV)	f_v	f_{org}
1.90	0.55	1

A NPs volume fraction of 0.55 is obtained, which is in agreement with absorbance and volume fraction reported in the literature on ITO NPs films of comparable thickness.[13] The surrounding medium is found to be mainly composed by organic compounds (oleic acid and 1,2-dichlorobenzene solvent), suggesting that the presence of air in the space between the NPs is negligible. The plasma frequency (ω_p) (1.9 eV) obtained in the fitting is comparable to the one obtained for the solution.

[13] Matsui et al., ACS Appl. Mater. Interfaces 2016, 8, 11749–11757 | Garcia et al., Nano Lett. 2011, 11, 4415–4420

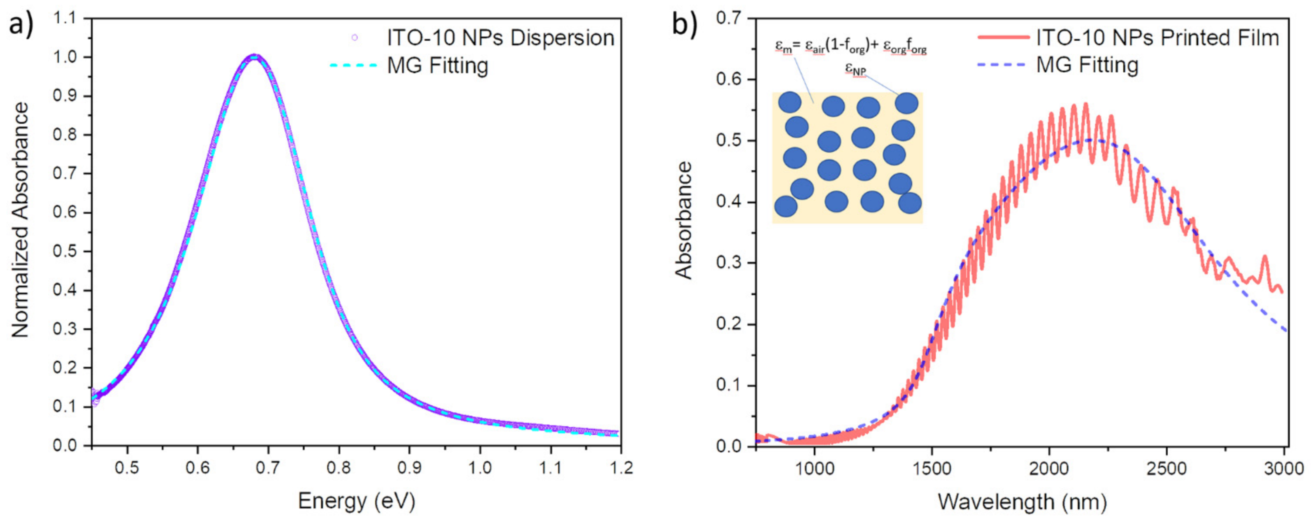

Figure S10 – a) Fit of normalized absorbance spectrum of ITO-10 dispersed in C_2Cl_4. b) Spectrum of printed ITO-10 NPs (25 1D), and related fitting performed using MG approach. In inset, a sketch of the model employed is depicted, with NPs of dielectric function ε_{NP} immersed in a medium with dielectric constant ε_m.

Further considerations on the validity of MG approach to fit printed ITO NPs. The main limitation of MG approach is that it neglects the inter-particle interactions, which may be important at very high packing density. Nevertheless, this approach is often used also at high volume fraction also for dense films of ITO NPs (volume fractions larger than 0.33).[14] However, attention should be taken when using the effective medium approach if the particles are very close or touch each other. A work by Reich and Shklovskii[15] explore the divergence of MG permittivity from the experimental data in arrays of densely packed NPs, depending on the ratio $\varepsilon_{NP}/\varepsilon_m$, and on the NP size compared to their distance. According to this work, if $\varepsilon_{NP}/\varepsilon_m<30$ MG theory predicts the dielectric function with high accuracy if the ratio between inter-particle separation (s) and NP size (a) is less than 0.025. In our work the average size of the ITO NPs is 10 nm, while the volume ratio between ITO and the organic coating layer is 60:40 as determined by thermogravimetric experiments. This volume ratio, considering the size of the inorganic core, is consistent with an organic shell with thickness of at least 1 nm. The distance between two NPs (s) is thus at least 2 nm (the sum of the two organic shells), thus s/a=0.4 that is greater than 0.025. This gives us a certain degree of confidence in using MG approach. Moreover, several works have pointed out the existence of a depletion layer in ITO NPs,[16] i.e. a layer close to the surface where the carrier density is considerably reduced due to

[14] Mendelsberg, R. J.; Garcia, G.; Milliron, D. J. Extracting Reliable Electronic Properties from Transmission Spectra of Indium Tin Oxide Thin Films and Nanocrystal Films by Careful Application of the Drude Theory. Journal of Applied Physics 2012, 111 (6), 063515. | Zandi, O.; Agrawal, A.; Shearer, A. B.; Reimnitz, L. C.; Dahlman, C. J.; Staller, C. M.; Milliron, D. J. Impacts of Surface Depletion on the Plasmonic Properties of Doped Semiconductor Nanocrystals. Nat. Mater. 2018, 17 (8), 710–717. | Garcia, G.; Buonsanti, R.; Runnerstrom, E. L.; Mendelsberg, R. J.; Llordes, A.; Anders, A.; Richardson, T. J.; Milliron, D. J. Dynamically Modulating the Surface Plasmon Resonance of Doped Semiconductor Nanocrystals. Nano Lett. 2011, 11 (10), 4415–4420.

[15] Reich, K. V.; Shklovskii, B. I. Dielectric Constant and Charging Energy in Array of Touching Nanocrystals. Appl. Phys. Lett. 2016, 108 (11), 113104.

[16] Gibbs, S. L.; Staller, C. M.; Milliron, D. J. Surface Depletion Layers in Plasmonic Metal Oxide Nanocrystals. Acc. Chem. Res. 2019, 52 (9), 2516–2524. | Agrawal, A.; Kriegel, I.; Runnerstrom, E. L.; Scotognella, F.; Llordes, A.; Milliron, D. J. Rationalizing the Impact of Surface Depletion on Electrochemical Modulation of Plasmon Resonance Absorption in Metal Oxide Nanocrystals. ACS Photonics 2018, 5 (5), 2044–2050. | Staller, C. M.; Robinson, Z. L.; Agrawal, A.; Gibbs, S. L.; Greenberg, B. L.; Lounis, S. D.; Kortshagen, U. R.;

surface defects. Such depletion layer would further screen the interaction between the active NP cores of two neighbour NPs. In the work by Zandi et al., MG approach is also applied to fit a film of uncoated ITO NPs deposited on a transparent electrode, making similar considerations about the validity of MG approach.

Milliron, D. J. Tuning Nanocrystal Surface Depletion by Controlling Dopant Distribution as a Route Toward Enhanced Film Conductivity. Nano Lett. 2018, 18 (5), 2870–2878.

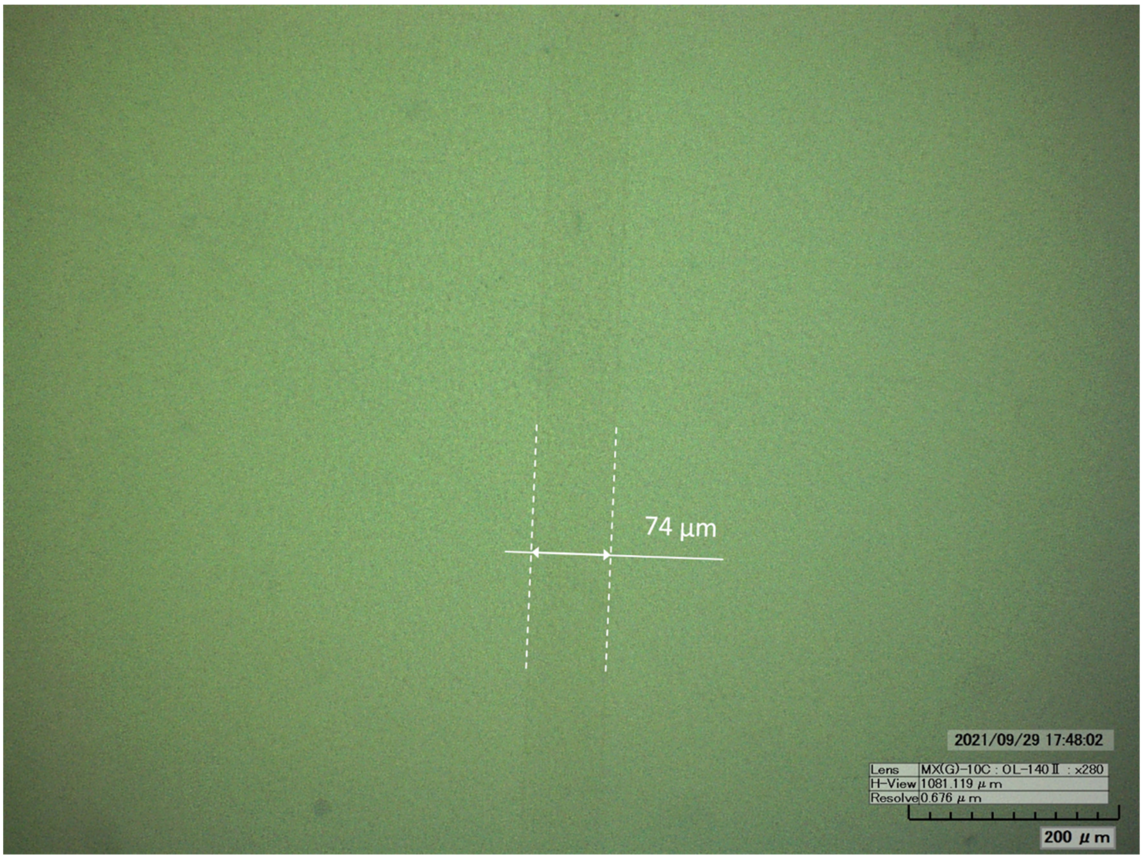

Figure S11 –Thinner single line obtained by ink jet printing, corresponding to the ITO ink printing resolution limit achievable with available instrumentation (Dimatix DMP-2850 printer).

Thermal simulations

Details on thermal simulation. A time-dependent study has been performed by using COMSOL Multiphysics 5.6, considering Heat Transfer in Solids. This latter allows the modelling of thermal conduction by solving the following governing equations:

$$\rho C_p \frac{\partial T}{\partial t} + \rho C_p \mathbf{u} \cdot \nabla T + \nabla \cdot \mathbf{q} = Q \quad \text{(S7)}$$

$$\mathbf{q} = -k\nabla T \quad \text{(S8)}$$

In (1) ρ is the density, C_p is the specific heat capacity at constant stress, T is the absolute temperature, $\mathbf{u}$ is the velocity vector of translational motion, $\mathbf{q}$ is the heat flux by conduction, and Q contains additional heat sources that, however, were not directly considered in this model. Equation (2) represents the Fourier's Law of heat conduction that states that the conductive heat flux, $\mathbf{q}$, is proportional to the temperature gradient where the coefficient of proportionality, k, is the thermal conductivity and takes a positive value meaning that heat flows from regions of high temperature to low temperature.

In such a model the other fundamental part was the heat exchange derived from the radiation that we considered by imposing the Stefan-Boltzmann equation on the entire domain:

$$k\nabla T = \varepsilon\sigma(T_{amb}^4 - T^4) \quad \text{(S9)}$$

in which σ is the Stefan-Boltzmann constant equal to $5.67 \cdot 10^{-8}$ W/m^2K^4 while ε represents the emissivity of the bodies. The heat source was modelled as a heat flux applied on the ITO-NPs square as the following boundary condition:

$$k\nabla T = \frac{P_0}{A} \quad \text{(S10)}$$

where the term P_0/A accounts for the inward heat flux derived from the applied power per surface area. The value of P_0/A flux has been obtained convoluting the experimentally measured absorbance of the ITO-10 25D 1L printed sample (as in Supporting Figure S8) with theoretical emission spectra of IR lamp, calibrated on nominal power value in the condition used for thermal experimental measurements (see next section for details). Additional thermal flux (P_{sub}/A) was also applied to the whole simulated surface to take into account the absorption of the PEN substrate.

A second boundary condition imposed on the whole domain was the Neumann boundary condition in order to consider the convective cooling due to the interaction between the sample and the air (h=2.5 $W/(m^2K)$):

$$k\nabla T = h(T_{ext} - T) \quad \text{(S11)}$$

In the model we imposed T_{ext}=T_{amb}=26.2 °C.

The used parameters have been defined as in the Supporting Figure S12. The only adjusted parameter was P_{sub}/A=18 W/m^2, obtained as best fitting by a series of preliminary simulations made only on substrate and compared with experimental data. This model, despite the simplicity, shows a very nice agreement with the experimental data, as it is possible to evaluate in Supporting Figure

S12. Result of simulation over thickness and time, in term of thermal profile distribution, are reported in Figure 13 for some selected values.

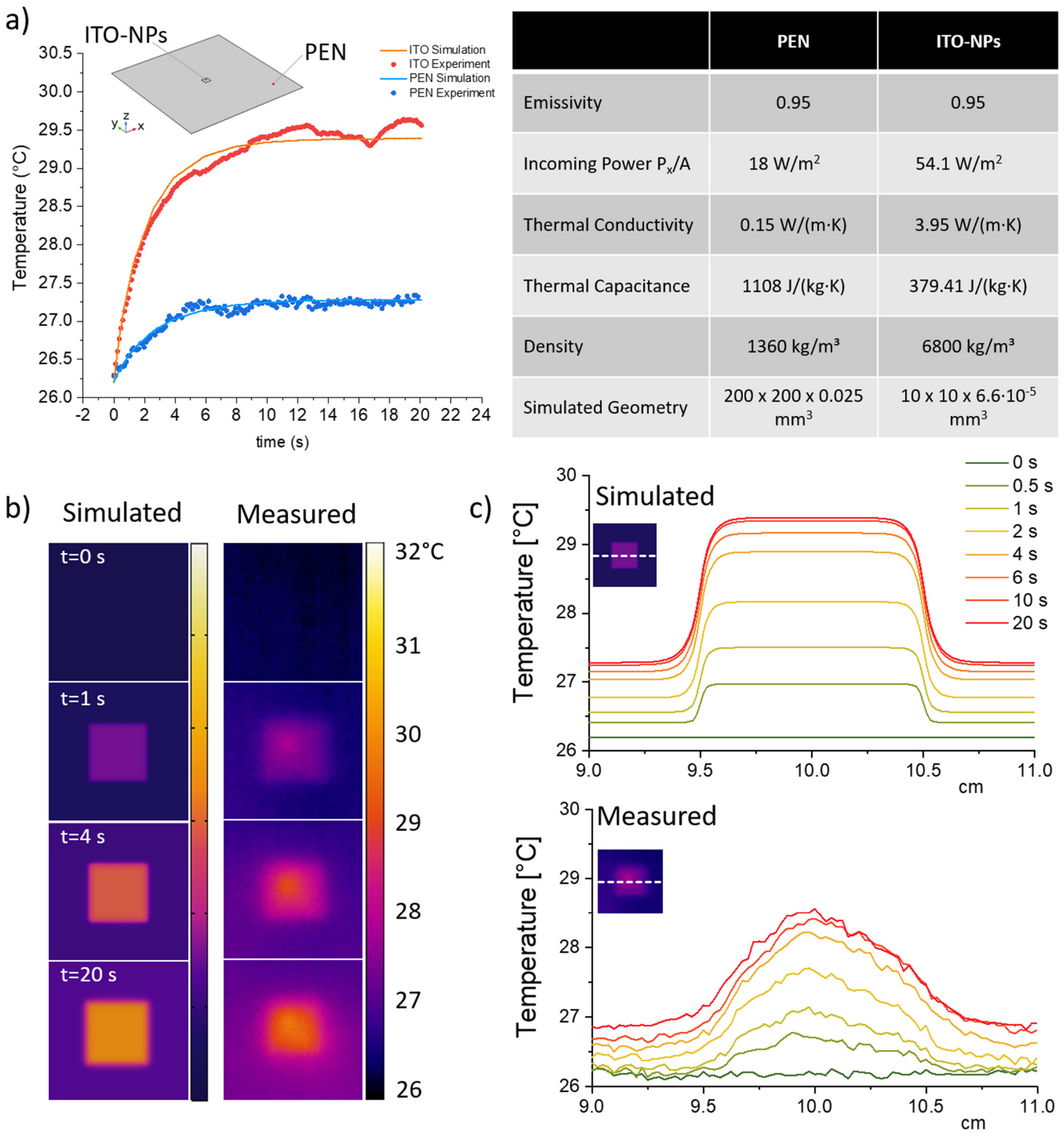

	PEN	ITO-NPs
Emissivity	0.95	0.95
Incoming Power P_x/A	18 W/m^2	54.1 W/m^2
Thermal Conductivity	0.15 W/(m·K)	3.95 W/(m·K)
Thermal Capacitance	1108 J/(kg·K)	379.41 J/(kg·K)
Density	1360 kg/m^3	6800 kg/m^3
Simulated Geometry	200 x 200 x 0.025 mm^3	10 x 10 x $6.6 \cdot 10^{-5}$ mm^3

Figure S12 – Simulation of dynamic heating of ITO-10 square sample (25DS single layer) printed on PEN substrate, compared with acquired data (as in main text **Figure 4b**). a) Geometry of model used for simulation and thermal dynamics of heating (simulation vs experiment comparison) in representative points. b) Thermal image acquired by IR camera (right) compared with simulated thermal one (left) at different time instants. c) Thermal profile along x-axes extracted by acquired by thermal images (bottom) and from simulations (top). In general, the simulated model shows a good matching with the experimental observations. Simulation parameters are reported in the table.

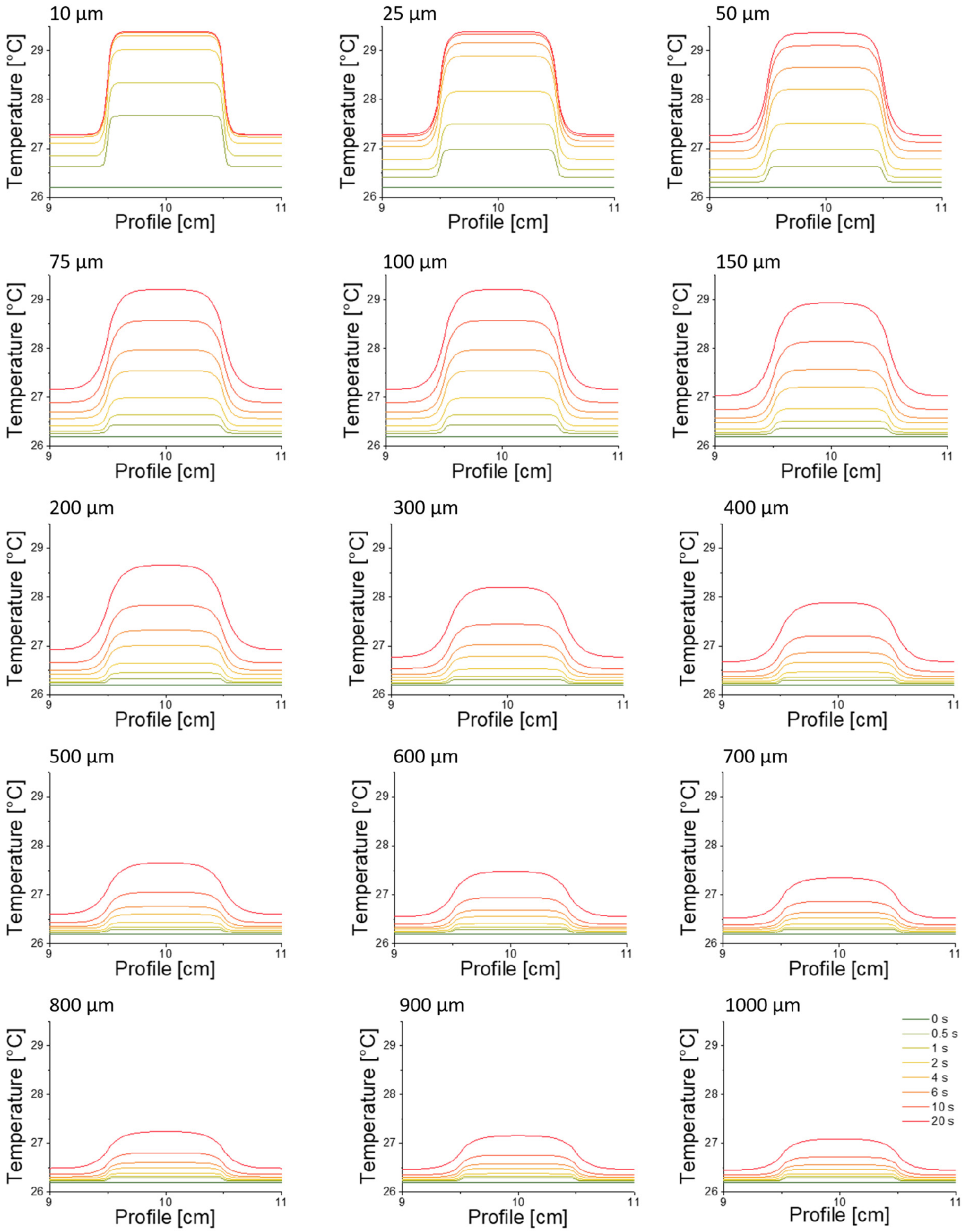

Figure S13 – Simulation of thermal profile distribution on various samples with different substrate thicknesses (10 - 1000 µm) at different instants (0 - 20 s).

Power flux estimation. The value of P_0/A flux (the absorbed power flux in the unity of surface), used to simulate the absorption of the thin film of ITO NPs, has been derived as the convolution of the experimentally measured absorbance of the ITO-10 25D 1L printed sample, and the theoretical emission spectra of the IR lamp, over the wavelength range of interest.

In particular, the spectral radiance of a black body $\boldsymbol{W_\lambda}$ (spectral emissive power per unit area and wavelength), function of absolute temperature $\boldsymbol{T}$ and wavelength $\boldsymbol{\lambda}$, is given by:

$$W_\lambda = \frac{C_1}{\lambda^5}\frac{1}{e^{\frac{C_2}{\lambda T}}-1} \tag{S12}$$

where C_1 and C_2 are constant are respectively given by

$$C_1 = 2\pi c^2 h = 3.75 \cdot 10^{-16}\, W \cdot m^2 \tag{S13}$$

$$C_2 = hc/k = 1.44 \cdot 10^{-2}\, m \cdot K \tag{S14}$$

with $\boldsymbol{h}$ Planck's constant, $\boldsymbol{c}$ the speed of light and $\boldsymbol{k}$ the Boltzmann constant.

Given the fraction of incident light which is effectively absorbed at certain wavelength defined as $\boldsymbol{A(\lambda)}$=[1-$T(\lambda)$], where $T(\lambda)$ is the transmittance (neglecting the reflectance contribution as working approximation), the value of P_0/A value can be calculated as follow:

$$\frac{P_0}{A} = E_0 \frac{\int W_\lambda(\lambda,T_K)\cdot A(\lambda)\, d\lambda}{\int W_\lambda(\lambda,T_K)\, d\lambda} = 54.1\, W/m^2 \tag{S15}$$

Where $\boldsymbol{E_0}$ is the nominal lamp optical power flux arriving on the sample in selected conditions (corresponding to E_0= 210 W/m^2, evaluated for distance and position respectively 50 cm and 4 cm out of axis as in the experimental measures, performed with 100W IR lamp with typical temperature $\boldsymbol{T_K}$ = 2450 K.

In Supporting Figure S13 it is possible to see in comparison, the spectral radiance of the lamp $W_\lambda(T_K)$, $A(\lambda)$, its normalized product defined as $\chi(\lambda) = \frac{W_\lambda(\lambda,T_K)\cdot A(\lambda)}{\int W_\lambda(\lambda,T_K)\, d\lambda}$, and its integral $\int_{\lambda_0}^{\lambda} \chi(\lambda)\, d\lambda$. Integration has been performed in the λ range 0.3-10 µm (outside this range the contribution of the lamp irradiation is considered negligible).

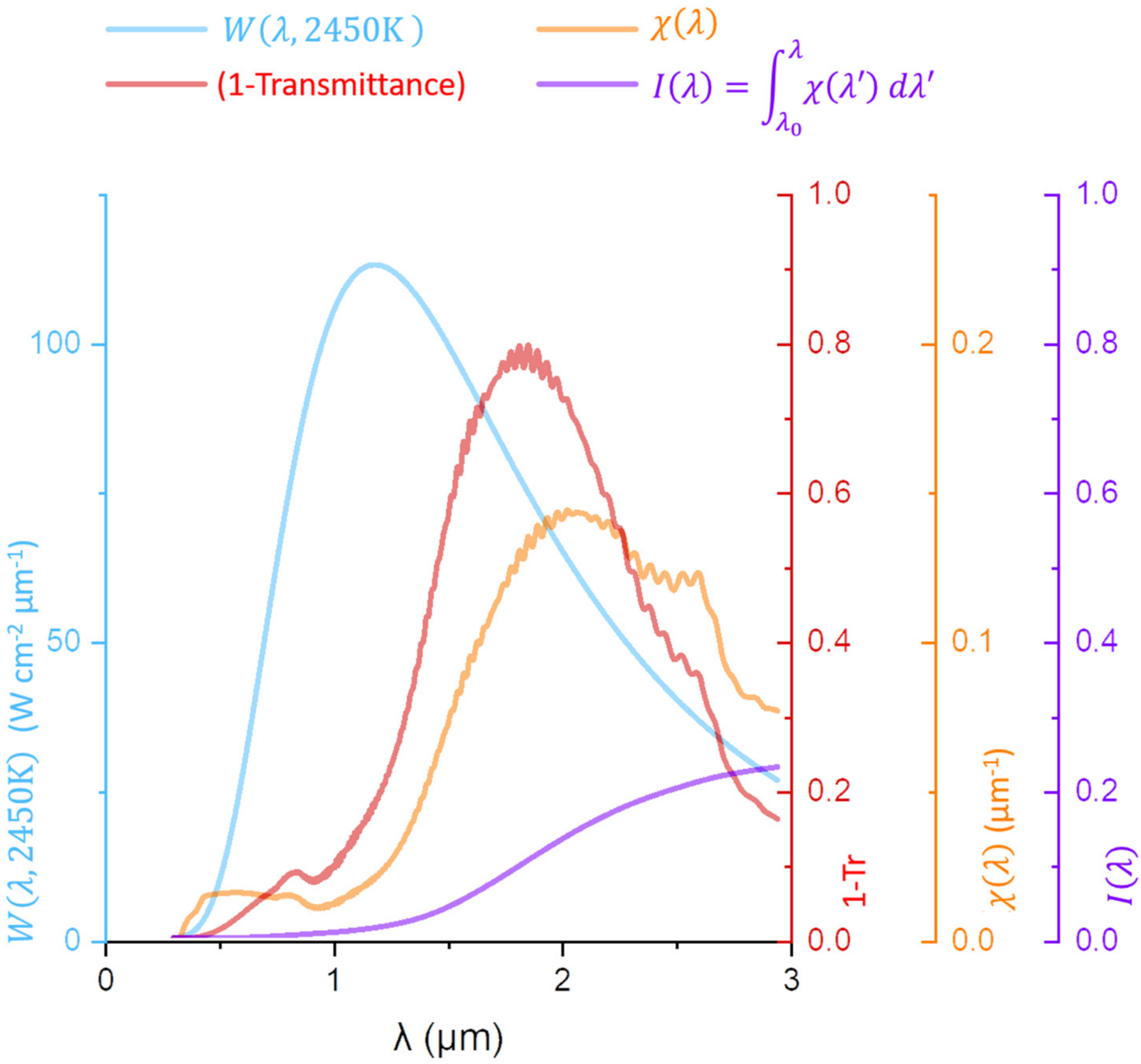

Figure S14 – Graph reporting in comparison spectral radiance of the lamp W_{λ} = W(λ, 2450K) (cyano curve), the fraction of incident light which is effectively absorbed *A(λ)* = (1 – Transmittance (λ)) (red curve), their normalised product $\chi(\lambda)$ (orange curve) and its integral over λ (violet curve).

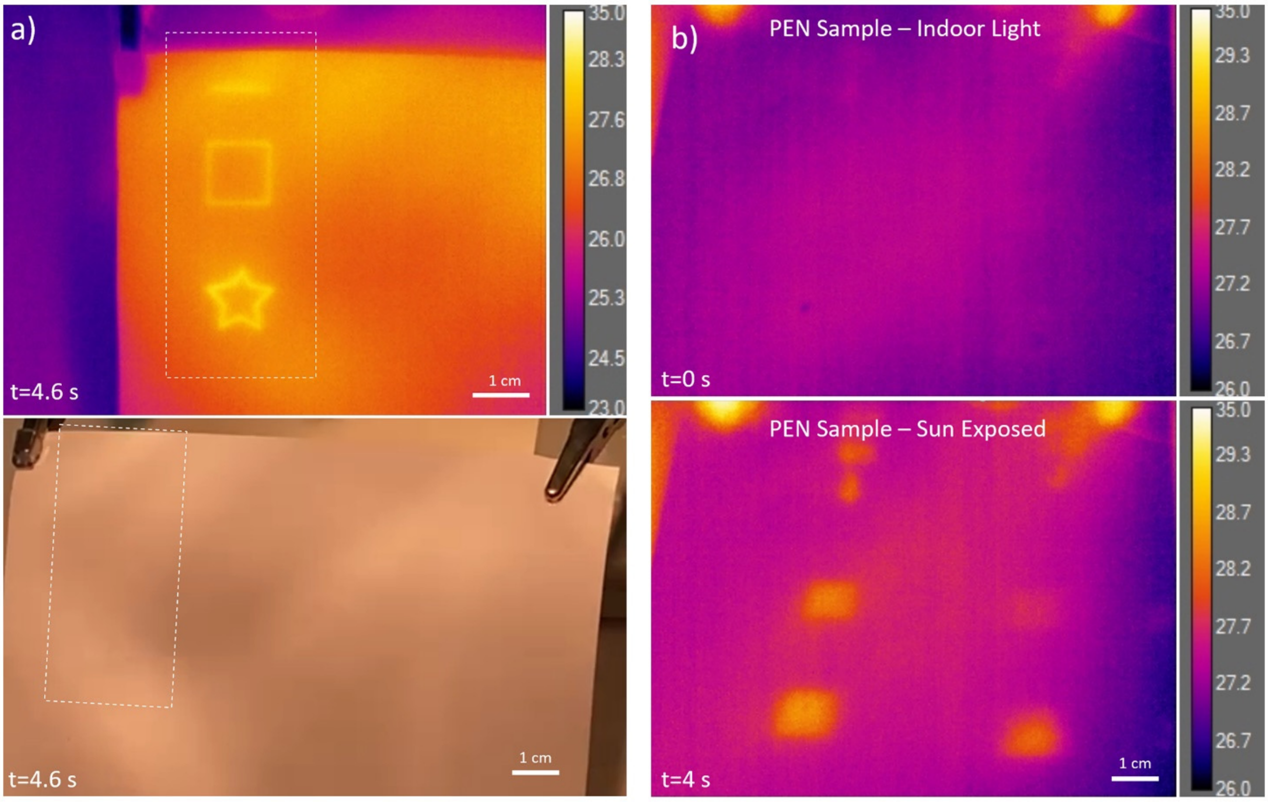

Figure S15 – Other examples of use of the ITO thermal ink. a) Example of ITO printed patterns on paper, thermal (up) and optical (down) image of the same sample under IR irradiation. b) Example of ITO printed square on PEN, when exposed to direct sunlight in indoor conditions (bottom), compared to the same sample not exposed (up).

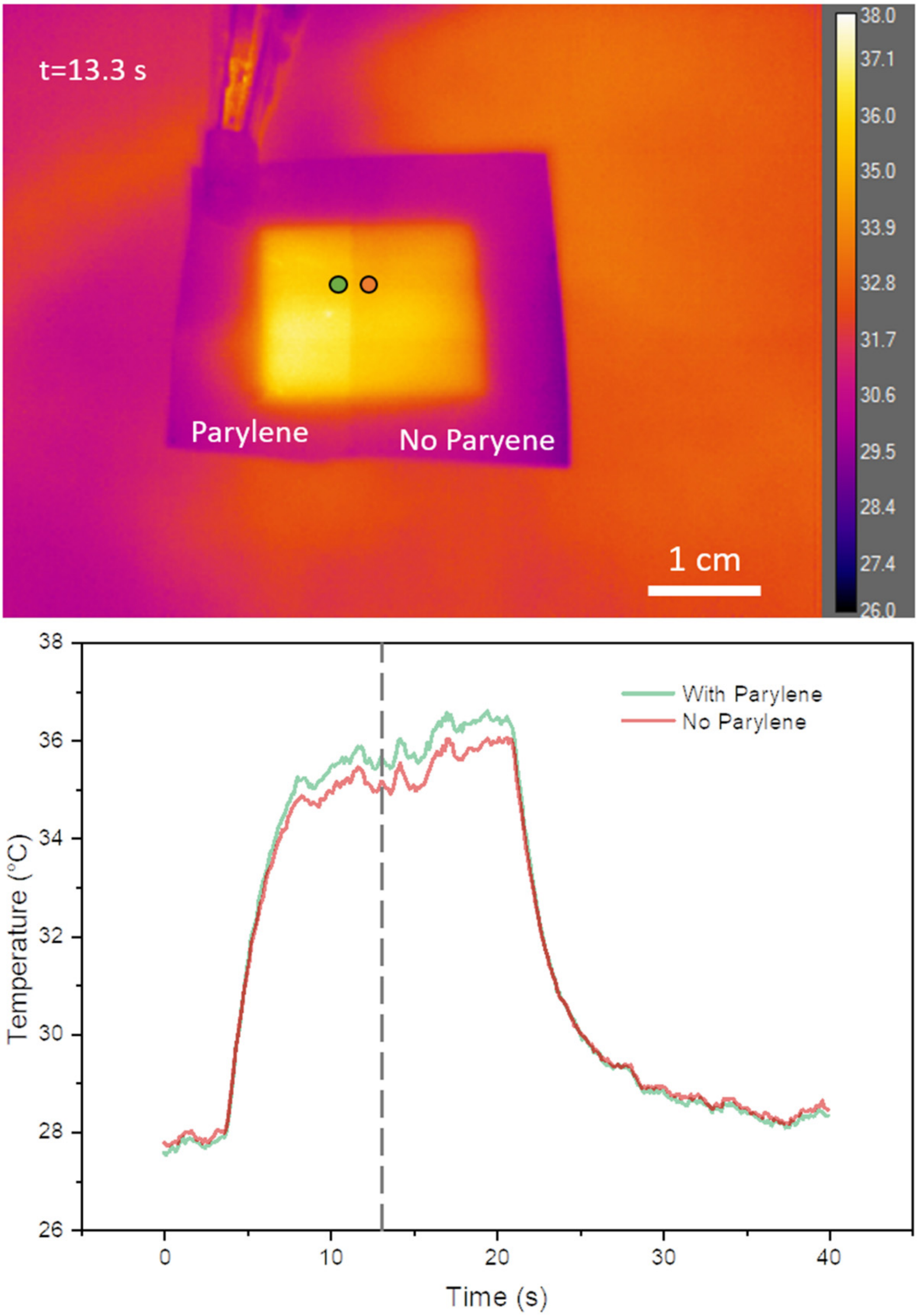

Figure S16 – Thermal response dynamics to IR irradiation of a sample half covered with a micrometric layer of parylene. The coloured dots represent the point in which the temperature is evaluated. As it is possible to observe, the part covered by parylene has reaches higher values of temperature respect to the uncovered one.
